# Neandertal cannibalism and Neandertal bones used as tools in Northern Europe

**DOI:** 10.1038/srep29005

**Published:** 2016-07-06

**Authors:** Hélène Rougier, Isabelle Crevecoeur, Cédric Beauval, Cosimo Posth, Damien Flas, Christoph Wißing, Anja Furtwängler, Mietje Germonpré, Asier Gómez-Olivencia, Patrick Semal, Johannes van der Plicht, Hervé Bocherens, Johannes Krause

**Affiliations:** 1 Department of Anthropology, California State University Northridge, 18111 Nordhoff St, Northridge, CA 91330-8244, USA; 2 Université de Bordeaux, CNRS, UMR 5199-PACEA, A3P, Allée Geoffroy Saint Hilaire, CS 50023, 33615 Pessac Cedex, France; 3 Archéosphère, 2 Rue des Noyers, 11500 Quirbajou, France; 4 Institute for Archaeological Sciences, Archaeo- and Palaeogenetics, University of Tübingen, Rümelinstr. 23, 72070 Tübingen, Germany; 5 Max Planck Institute for the Science of Human History, Khalaische Straße 10, 07745 Jena, Germany; 6 Laboratoire TRACES – UMR 5608, Université Toulouse Jean Jaurès, Maison de la Recherche, 5 Allée Antonio Machado, 31058 Toulouse Cedex 9, France; 7 Department of Geosciences, University of Tübingen, Hölderlinstr. 12, 72074 Tübingen, Germany; 8 Royal Belgian Institute of Natural Sciences, 29 Vautier St, 1000 Brussels, Belgium; 9 Departmento de Estratigrafía y Paleontología, Facultad de Ciencia y Tecnología, Euskal Herriko Unibertsitatea, UPV-EHU. Apdo. 644, 48080 Bilbao, Spain; 10 IKERBASQUE, Basque Foundation for Science, María Díaz de Haro 3, 48013 Bilbao, Spain; 11 UMR 7194 CNRS, Département de Préhistoire, Muséum national d’Histoire naturelle, Musée de l’Homme, 17 Place du Trocadéro, 75016 Paris, France; 12 Centro Mixto UCM-ISCIII de Evolución y Comportamiento Humanos, Avda. Monforte de Lemos 5, 28029 Madrid, Spain; 13 Centre for Isotope Research, Groningen University, Nijenborgh 4, 9747 AG Groningen, Netherlands; 14 Faculty of Archaeology, Leiden University, PO Box 9514, 2300 RA Leiden, Netherlands; 15 Senckenberg Centre for Human Evolution and Palaeoenvironment, University of Tübingen, 72072 Tübingen, Germany

## Abstract

Almost 150 years after the first identification of Neandertal skeletal material, the cognitive and symbolic abilities of these populations remain a subject of intense debate. We present 99 new Neandertal remains from the Troisième caverne of Goyet (Belgium) dated to 40,500–45,500 calBP. The remains were identified through a multidisciplinary study that combines morphometrics, taphonomy, stable isotopes, radiocarbon dating and genetic analyses. The Goyet Neandertal bones show distinctive anthropogenic modifications, which provides clear evidence for butchery activities as well as four bones having been used for retouching stone tools. In addition to being the first site to have yielded multiple Neandertal bones used as retouchers, Goyet not only provides the first unambiguous evidence of Neandertal cannibalism in Northern Europe, but also highlights considerable diversity in mortuary behaviour among the region’s late Neandertal population in the period immediately preceding their disappearance.

Neandertal funerary practices remain at the forefront of palaeoanthropological research, generating heated debates following the revision of old data and new excavations at key sites such as La Chapelle-aux-Saints^1^,^2^, Roc de Marsal^3^, Saint-Césaire^4^ and La Ferrassie^5^. More generally, attention has focused on the variability of Neandertal mortuary practices to evaluate their cognitive and symbolic implications, especially as they may provide insights concerning the social systems of this fossil human group^6^. Neandertals are known to have buried their dead and are associated with mortuary behaviours that are often difficult to interpret in Palaeolithic contexts. The site of Krapina (Croatia) is an instructive example in this sense. Evidence for cannibalism was first proposed for this site as early as 1901^7^ based on the fragmentation and traces of burning from a large collection of early Neandertal remains. This evidence has since been disputed by proponents of alternative explanations for the human bone modifications who argue for natural processes while others maintain that the anthropogenic manipulations are best interpreted in the context of secondary burials^8^. Several studies dedicated to cannibalism have proposed that securely identifying anthropogenic modifications related to this practice should incorporate evidence for the similar treatment of both faunal and human remains in the interest of extracting nutrients^9^,^10^,^11^. In addition to Gran Dolina (level TD6; Early Pleistocene) in Spain, which has produced the earliest undisputed evidence for cannibalism^12^, further examples have also been documented at several Western European Neandertal sites, including El Sidrón and Zafarraya^13^,^14^ in Spain, and Moula-Guercy and Les Pradelles^15^,^16^ in France.

Here we provide new data on the diversity of Neandertal mortuary behaviour, focusing on a small area of their known range, Northern Europe, during Marine Isotope Stage (MIS) 3 (ca. 60–30 thousand years ago), in order to identify small-scale processes during this short period that witnessed the disappearance of the Neandertals^17^. We present 99 new Neandertal remains recently identified among the collections from the Troisième caverne of Goyet (Belgium), some of which exhibit anthropogenic modifications, and discuss their implications.

The Troisième caverne (or “Third cave”) of Goyet, excavated in the latter half of the 19th and beginning of the 20th century, and again at the end of the 1990s^18^, is part of a large cave system located in the Mosan Basin (Supplementary Fig. S1). The most extensive excavations were carried out by Edouard Dupont in 1868, who described five “fauna-bearing levels” (FBL; ref. 19; Supplementary Note S1). The Troisième caverne yielded a rich archaeological sequence with Middle and Upper Palaeolithic deposits containing Mousterian, Lincombian-Ranisian-Jerzmanowician (LRJ), Aurignacian, Gravettian and Magdalenian artefacts as well as Neolithic and historic period material^20^,^21^,^22^,^23^. Whether the Mousterian material derives from a single or multiple phases of occupation is currently impossible to discern (Supplementary Note S2). Unfortunately, the excavation methods did not meet today’s standards, and it appears that the levels described by Dupont actually represent a mix of material from different periods (e.g., ref. 24).

Several human remains from different levels were published by Dupont^19^ and Hamy^25^, although only a few figure in the Catalogue of Fossil Hominids^26^, all of which were attributed to the Magdalenian. In 2004, we identified both a Neandertal mandible fragment and an isolated tooth among the human material recovered by Dupont from the Troisième caverne and currently housed at the Royal Belgian Institute of Natural Sciences (RBINS)^27^, making Goyet one of the few Northern European sites north of 50° N to have yielded MIS 3 Neandertal remains (Supplementary Note S1 and Supplementary Fig. S1).

## Results

### Identification of new Neandertal remains at Goyet and their biogeochemical characterization

**Figure 1:**
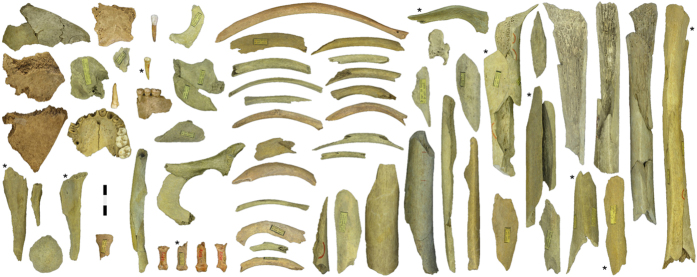
Neandertal remains from the Troisième caverne of Goyet (Belgium). *Designates the specimens that have been directly dated. Scale = 3 cm.

The reanalysis of the Goyet material comprised (i) the revision of the human skeletal material, (ii) systematic sorting of the faunal collections to check for unidentified human remains (Supplementary Fig. S2), and (iii) a multidisciplinary study of the human remains and their context. Two-hundred and eighty three human remains were identified from different periods, including 96 bone specimens and three isolated teeth identifiable as Neandertal (Supplementary Table S1 and Supplementary Notes S3, S4 and S5). A good number (n = 47) of the bone specimens refit, reducing the total number of isolated Neandertal remains to 64 (Fig. 1 and Supplementary Table S2), of which 10 were directly radiocarbon (^14^C) dated, 15 were sampled for stable isotope analyses, and 10 for DNA extraction (Table 1 and Supplementary Table S3). Based on their morphology and morphometric characteristics, developmental stage and side for paired elements, as well as the successful recovery of endogenous mitochondrial DNA (mtDNA) sequences, the minimum number of individuals (MNI) represented by the Goyet sample is estimated at five (four adolescents/adults and one child represented by a single tooth; Supplementary Note S5 and Supplementary Fig. S3). Although the Neandertal sample includes cranial and postcranial elements (Fig. 1), with long bones best represented and extremities mostly absent, the minimum number of elements (MNE = 35) demonstrates a very low overall skeletal representation. The best represented elements are, in decreasing order, the tibia (six of the eight tibias expected for four adolescents/adults, 75% representation), femur and cranium (50%), humerus and mandible (25%; Supplementary Table S4).

Chemical elemental analyses performed together with stable isotope analyses were used to assess collagen preservation in preparation of ^14^C dating (see Methods). The ecology of the Goyet Neandertals was also investigated using δ^13^C and δ^15^N isotope composition of bone collagen^28^. Direct ^14^C dates obtained from the newly identified skeletal material place the Goyet Neandertals to ca. 40.5–45.5 ky calBP. However, when the youngest ages, which likely reflect undetected bone collagen contamination, are excluded (Supplementary Note S6), we cannot rule out the possibility that the Goyet Neandertals represent a single chronological group dating to ca. 44–45.5 ky calBP. Although this appears the most parsimonious hypothesis when individual bone associations, taphonomic aspects and similar anthropogenic modifications observed across the sample are taken into account, we retain the conservative range of ca. 40.5–45.5 ky calBP for the Goyet Neandertals in the absence of definitive evidence.

**Figure 2:**
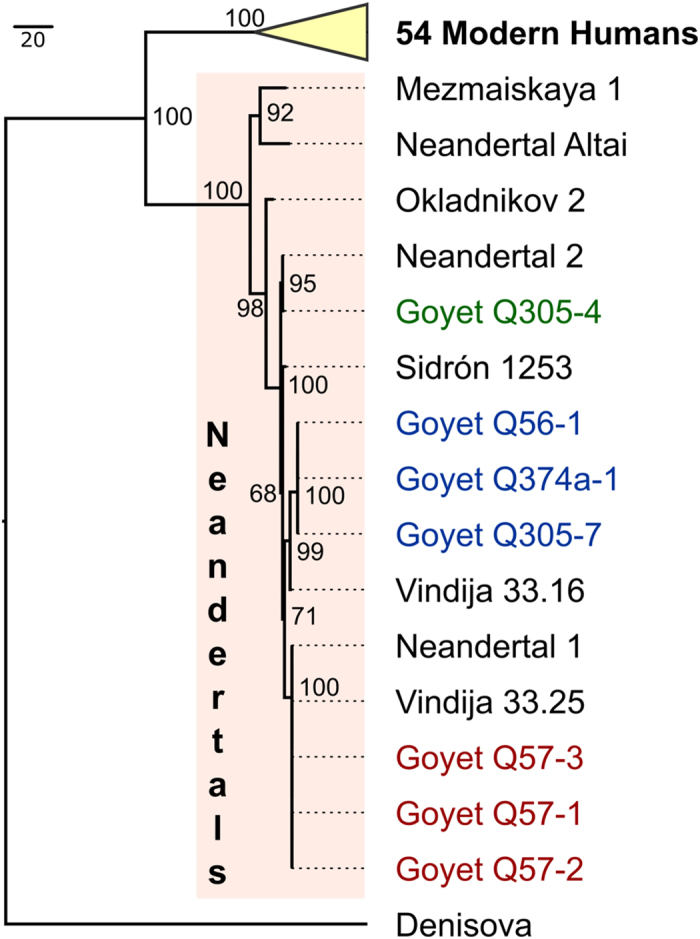
Maximum parsimony tree for the seven analysed Goyet samples that produced complete or almost complete mitochondrial genomes compared to 63 published modern human, Neandertal and Denisovan mtDNAs. Numbers at the main branch nodes represent bootstrap values after 1,000 iterations.

Out of the 10 samples processed for genetic analysis, seven show three distinct complete or almost complete mtDNA lineages (noted 1–3 in Table 1). The newly reconstructed mtDNAs from Goyet were compared with the mtDNA of 54 modern humans, eight previously sequenced Neandertals and one Denisovan individual^29^,^30^,^31^,^32^,^33^,^34^. Phylogenetic relationships were assessed using maximum parsimony and maximum likelihood trees (Fig. 2 and Supplementary Fig. S4), confirming the analysed specimens to fall within the known diversity of Neandertal mtDNA. The Goyet Neandertal mtDNAs appear most closely related to late Neandertal mtDNAs from Central and Western Europe, such as those from the Neandertal type-site (Germany), El Sidrón (Spain) and Vindija (Croatia), which all show only modest genetic variation despite large geographic distances when compared to modern humans. As previously suggested^31^, this might reflect a low effective population size of Neandertals in general, and for the late Neandertals in particular.

### Taphonomic analysis of the Goyet Neandertal material and anthropogenic modifications

**Figure 3:**
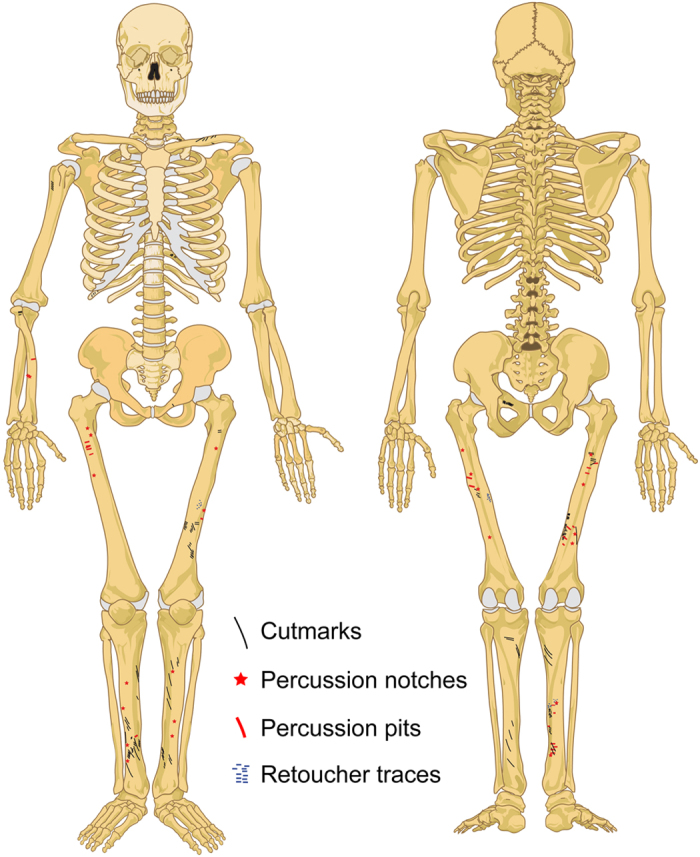
Overview of the anthropogenic modifications observed on the Neandertal remains from the Troisième caverne of Goyet (Belgium). See Supplementary Fig. S8 for individual Neandertal bones with anthropogenic modifications. Skeleton diagrams modified from https://en.wikipedia.org/wiki/File:Human_skeleton_front_en.svg and https://en.wikipedia.org/wiki/File:Human_skeleton_back_en.svg using Adobe Illustrator CS4 v. 14.0.0.

**Figure 4:**
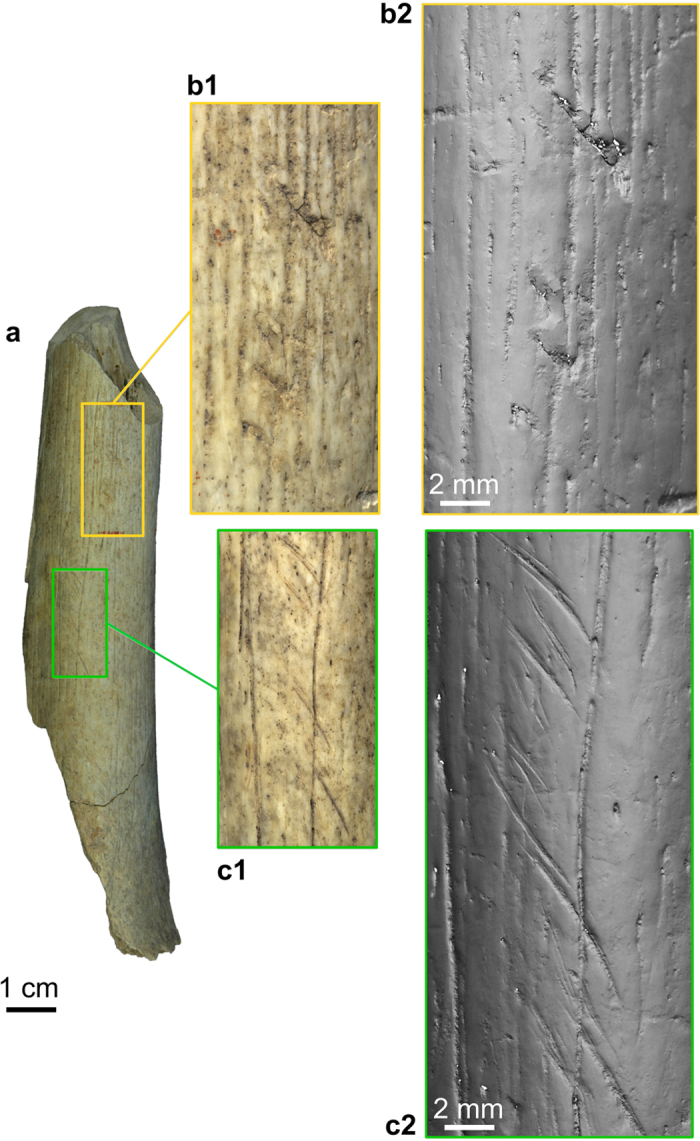
Retouching marks (b1,b2) and cutmarks (c1,c2) present on the Goyet Neandertal bones (example of femur III). (**a**) femur III in anterior view; (**b1**,**c1**) close-up photos; (**b2**,**c2**) images obtained using a minidome (see Methods).

**Figure 5:**
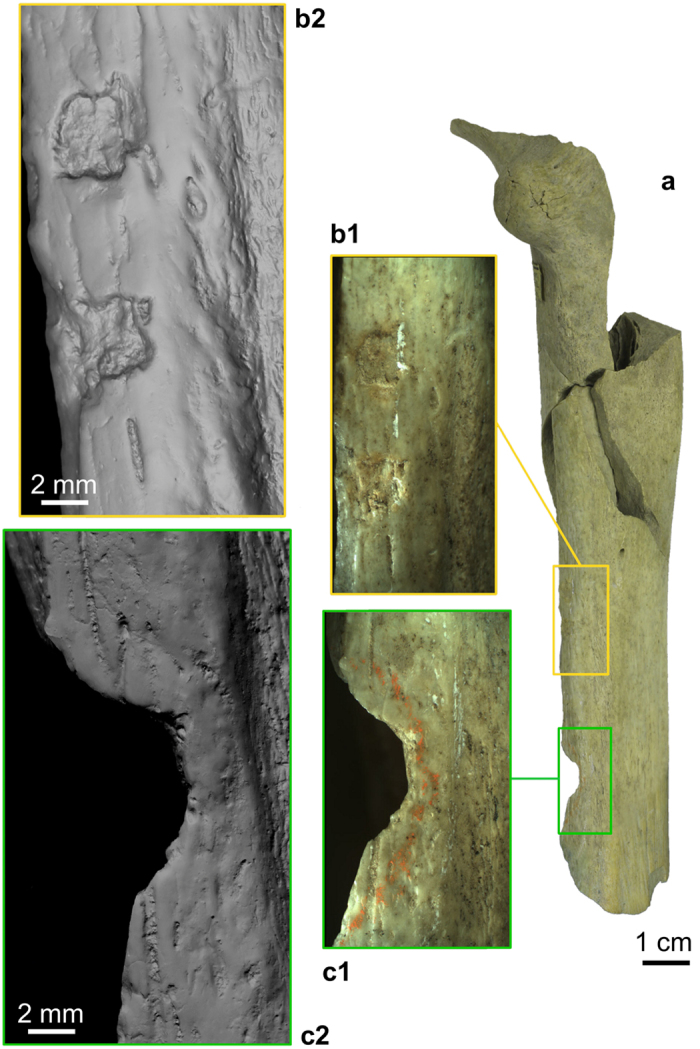
Percussion pits (b1,b2) and percussion notch (c1,c2) present on the Goyet Neandertal bones (example of femur I). (**a**) femur I in posterior view; (**b1**,**c1**) close-up photos; (**b2**,**c2**) images obtained using a minidome (see Methods).

Overall, the Neandertal remains are highly fragmented. Forty-nine percent of the bone specimens (47 out of 96) were refit to at least one other, with the number of specimens per refit set ranging from 2 to 8 (tibia I; Supplementary Fig. S5). Several examples of refits between levels 1 through 3 were also identified. None of the Neandertal bones are complete, although the proximal extremity of a hand phalanx (2878–37) is only slightly eroded (Fig. 1). Cortical surfaces are well preserved and exhibit limited post-depositional modifications. Most long bones fractures involve green breaks, as indicated by smooth margins and spiral fractures^35^. Traces of peeling may also provide evidence for the fresh bone fracture of a cranial fragment and several ribs (ref. 11; Supplementary Fig. S6). Although bears can produce such traces^36^, the presence of cutmarks on several ribs (see below) suggests that the most parsimonious hypothesis is that they are anthropogenic. Traces of human chewing^37^,^38^ are also suspected on the Neandertal phalanges but are inconclusive (Supplementary Fig. S6). The numerous unambiguous anthropogenic marks on the Goyet Neandertal remains can be attributed to three categories of bone surface modifications (Figs 3, 4, 5, Table 2, and Supplementary Figs S7 and S8):

1. Cutmarks. Nearly a third of the Neandertal specimens bear cutmarks. The locations of the limited number of cutmarks observed on the upper limb may indicate disarticulation whereas those on the lower limb are consistent with defleshing. Several cutmarks on the internal and external surfaces of the ribs may be connected to evisceration, dismemberment of the thoracic cage and removal of the thoracic muscles. An additional cutmark on the medial side of the mandible, close to the mandibular condyle, appears consistent with dismemberment.
2. Two types of percussion marks (notches and pits) were identified. Observed only on a single radius alongside several femurs and tibias, notches are likely connected to the fracturing of fresh diaphyses and marrow extraction. Percussion pits are common and probably indicate failed attempts at fracturing bones. Both percussion notches and pits were also identified on eight bones (e.g. femur I, Fig. 5).
3. Retouching marks. These marks, found on a femur and three tibias (Supplementary Figs S9–S12), result from retouching the edges of stone tools. The fact that none of the affected areas overlap on adjacent fragments suggests the bones to probably have first been marrow cracked. Femur III shows two retouching zones on the anterior and postero-medial surfaces, both located at mid-shaft. Interestingly, the traces found on the tibias are located in the same areas of the shaft on all three bones (posterior or postero-medial surface at mid-shaft). The retouchers are made on four different Neandertal bones that represent at least three of the four adolescent/adult Neandertal individuals (Supplementary Note S5).

While animal bone retouchers are common in European Middle Palaeolithic contexts (e.g., refs 39, 40, 41), Goyet is one of only four sites (Krapina in Croatia^42^, La Quina and Les Pradelles in France^43^,^16^) to have yielded retouchers on Neandertal skeletal elements and the sole to have produced multiple examples (Table 3). At Krapina and Les Pradelles, femur shaft fragments were used as retouchers, whereas the La Quina example is on a parietal fragment. According to the criteria proposed by Mallye *et al*.^40^, the blanks used for the Goyet retouchers made on Neandertal bones were most likely green due to the absence of scaled areas, and in addition, two of the five retoucher areas exhibit concentrated and superposed marks which imply prolonged use. The rectilinear morphology of the marks also supports the use of the bones for retouching flint flakes, the most common raw material found at Goyet.

### Comparative taphonomic analysis of the fauna from the Troisième caverne

Due to the large size of the Goyet faunal collection (>30,000 specimens), only a sample from Dupont’s excavation was examined (see Methods; Supplementary Fig. S2 and Supplementary Table S5). The skeletal material analysed corresponds mostly to long bone shaft fragments from various species that were mixed together within the collection and did not appear to have been previously sorted. We focused on remains from levels 3 and 2, which yielded the Neandertal remains, and on material from the same storage trays containing the human remains in order to have an overview of the associated faunal spectrum and assess food procurement and management strategies. Horse and reindeer are by far the most frequent species in the studied assemblage (86% of the 1,556 identified specimens; Supplementary Table S5). No rodent toothmarks were observed, carnivore remains are relatively sparse and carnivore damage is extremely rare on the Neandertal, horse and reindeer remains (Table 2), indicating carnivores to have had limited access to the bone material.

Anatomical profiles reveal numerous similarities between the Neandertal sample on one hand and horse and reindeer on the other (Supplementary Table S6 and Supplementary Fig. S13). The tibia is the most abundant element of all three species, whereas the axial skeleton and extremities of the forelimb and hindlimb are poorly represented. Bones of the hindlimb are better represented for all three species compared to forelimb elements, this is especially the case with the Neandertal material. The only notable difference between the faunal and Neandertal remains is the high representation of cranial elements for the latter. Unfortunately, the absence of contextual data precludes an analysis of the spatial distribution of both the faunal and Neandertal remains within the Troisième caverne.

The most intensely processed Neandertal elements are femurs and tibias (Supplementary Fig. S7), which are also the bones with the highest nutritional content (meat and marrow). The same pattern was documented for horse and reindeer bones. Overall, anthropogenic marks on the Neandertal remains match those most commonly recorded on the faunal material (Supplementary Figs S14–S16). All three taxa were intensively exploited, exhibiting evidence of skinning, filleting, disarticulation and marrow extraction. However, the Neandertal remains stand out as they show a high number of percussion pits (Table 2), which may be linked to the thick cortical structure of Neandertal long bones. Although the Neandertal remains show no traces of burning, the possibility that they may have been roasted or boiled cannot be excluded. The high number of cutmarks and the fact that DNA could be successfully extracted are, however, inconsistent with this possibility^44^,^45^,^46^. Lastly, similar to what has been noted at other sites^40^,^41^,^47^, the Neandertal retouchers are made on fragments of dense bones with comparable mechanical properties to the horse and reindeer bones. At Goyet, as at several French Middle Palaeolithic sites, large bone fragments of medium and large-sized animals were selected^40^,^41^,^48^,^49^,^50^,^51^. Among the Goyet Neandertal material, the largest and thickest fragments were also selected, as was the case at Les Pradelles^16^ and Krapina^42^. Interestingly, a femur and tibias of cave bears were also among the retoucher blanks selected by Neandertals at Scladina^52^.

The observed patterns of faunal exploitation can be interpreted as the selective transport of meat and marrow rich elements to the site that were subsequently intensively processed. However, this apparent pattern may reflect a collection bias favoring the largest and most easily identifiable fragments. Similarities in anthropogenic marks observed on the Neandertal, horse and reindeer bones do, however, suggest similar processing and consumption patterns for all three species.

## Discussion

Our results show that the Neandertals from the Troisième caverne of Goyet were butchered, with the hypothesis of their exploitation as food sources the most parsimonious explanation for the observed bone surface modifications. Goyet provides the first unambiguous evidence of Neandertal cannibalism in Northern Europe and given the dates obtained on the Neandertal remains, it is most likely that they were processed by their fellow Neandertals as no modern humans are known to have been in the region at the time^17^,^23^. However, the available data make it impossible to determine whether the modifications observed on the Neandertal skeletal material represent symbolic practices or simply result from the processing of immediately available sources of food. In addition, Goyet is the first site to have yielded multiple Neandertal bone retouchers. It has been proposed that Middle Palaeolithic retoucher blanks were by-products of the processing of carcasses for food consumption^40^,^41^, which may have been selected to be re-used^51^. The data at hand do not allow us to propose a different scenario for the Goyet retouchers made on Neandertal bones. However, the freshness of the blanks used suggests that Neandertals may have been aware that they were using human remains. Whether this was part of a symbolic activity or induced by a functional motivation cannot be attested, as was the case for the La Quina Neandertal retoucher^43^.

Although the Goyet late Neandertals date to 40.5–45.5 ky calBP, the lack of reliable contextual information makes it impossible to associate them with any of the technocomplexes from the site. However, coeval Mousterian assemblages are known from sites in the Mosan Basin, as at unit 1A of Scladina^53^, located only 5 km from Goyet, layer CI-8 of Walou Cave^54^, and layer II of Trou de l’Abîme at Couvin^55^ (Supplementary Note S2). While the LRJ is known from two sites in Belgium, Spy and Goyet, with its first appearance dated at other sites to around 43–44 ky calBP^23^,^56^), no reliable information is currently available for its regional chronology. Given the direct ^14^C dates obtained for the Goyet Neandertals, it is impossible to securely associate them with either the Mousterian occupation(s) or the LRJ.

In terms of the region’s late Neandertal mortuary practices, four sites within an approximately 250 km radius around Goyet produced Neandertal remains reliably dated to between 50–40 ky calBP (Supplementary Fig. S1). Interestingly, none of these sites produced evidence for the treatment of the corpse similar to that documented for Goyet. Two Belgian sites, Walou Cave and Trou de l’Abîme, produced, respectively, a premolar and a molar^55^,^57^. Although impossible to infer the behavioural signature represented by these remains, given their state of preservation it is highly unlikely that they involved funerary practices, including burial. In Germany, the Neandertal individuals from Feldhofer, including Neandertal 1, are possibly associated with the “Keilmesser group”, a late Middle Palaeolithic technocomplex^58^,^59^ unknown at Goyet (Supplementary Note S2). Neandertal 1 comprises elements of the cranial and postcranial skeleton of a single individual. Despite cutmarks on the cranium, clavicle and scapula, the long bones are intact and damage to still articulated skeletal elements during their recovery indicates that at least part of the skeleton may have originally been in anatomical connection^60^,^61^. Finally, at Spy, direct dates obtained on the two Neandertal adults place them within the current chronology of the LRJ^62^, although the association between the human remains and this technocomplex is uncertain due to the lack of contextual information. A recent reassessment of the Spy specimens and their context suggests that both individuals were buried^63^. And, it is worth noting that the most complete individual, Spy II, was originally described as a complete skeleton found in a contracted position. Moreover, the completeness of the skeleton and the absence of post-depositional alterations suggest the body to have been rapidly protected^63^.

Considerable diversity is evident in the mortuary behaviour of the late Neandertal populations of Northern Europe, possibly involving both primary and secondary deposits, alongside other types of practices, including cannibalism. Despite low genetic diversity amongst late Neandertal populations, the presence of various late Middle Palaeolithic technocomplexes, as well as the LRJ, nevertheless suggests significant behavioural variability amongst these groups in Northern Europe.

## Methods

### Collection assessment

The assessment of the Goyet collections included material housed at the RBINS and Royal Museums of Art and History (RMAH) in Brussels, which originate from the Troisième caverne, as well as collections from the *Grand Curtius* Museum (Liège), the *Cercle d’Histoire et d’Archéologie du Pays de Genappe* (Genappe), and the *Préhistosite de Ramioul* (Ramioul), whose origin is less secure. The Neandertal remains presented here were found among the first two collections only. The numbering system of the specimens and their origin are discussed in Supplementary Note S4.

### Taphonomic study

After determining the composition of the faunal assemblage sampled from Dupont’s collection (Supplementary Table S5), a total of 442 horse and 287 reindeer remains were observed using a monocular microscope (×10), as were all of the Goyet Neandertal remains. Taphonomic and anthropogenic modifications were recorded and drawn on anatomical charts (Supplementary Figs S8 and S14–S16). Cutmarks and trampling marks were distinguished according to their morphology and placement on bones^64^. Only unambiguous notches with a negative flake scar^65^,^66^ made on fresh bone^35^ and percussion pits (left by impact events after ref. 66) were recorded as percussion marks. The identified bone retouchers are all long bone diaphysis fragments that exhibit marks as described by Mallye *et al*.^40^. Finally, toothmarks were recorded using Binford’s typology^67^. Only pits and scores were observed. Some of these pits might have been produced by human chewing^38^,^67^,^68^,^69^ but they are not characteristic enough to definitely distinguish them from marks left by carnivores. Following Bello *et al*.^70^, the anthropogenic modifications recorded on the Neandertal remains were documented using drawings, close-up photographs and high-resolution imaging. The high-resolution images (Figs 4 and 5, and Supplementary Figs S8–S12) were obtained by using a minidome, a digital imaging device developed by VISICS at KULeuven (http://www.minidome.be). Based on the polynomial texture mapping technique, the dome consists of 260 LEDs and a single fixed camera, which captures an image with each LED individually lit. The results allow to display an object interactively under varying lighting to reveal all of the details of its surface. Additionally, 3D models of the retouchers made on Neandertal bones obtained using a white light 3D measurement system (http://www.mechscan.co.uk/) are available at http://virtualcollections.naturalsciences.be/virtual-collections/anthropology-prehistory/human-remains/goyet.

### Sample selection and preparation for isotopic and genetic analyses

All sampled specimens were untreated (glued or varnished), newly identified Neandertal bones, except for tooth 2878–2D (see Table 1). Specimens were scanned or μ-scanned and molded using DC-3481 silicone elastomer before sampling, with photos taken both before and after. Collagen was extracted at the Centre for Isotope Research of Groningen University (CIO, Netherlands) and the Biogeology working group of the Department of Geosciences of Tübingen University (Germany; see Supplementary Table S3). Radiocarbon dating was done at the CIO; stable isotope and genetic analyses were performed at Tübingen University.

### Isotope analyses

Collagen extraction at the CIO followed the procedure developed by Longin^71^, with additional chemical pretreatment using standard procedures^72^. Collagen extraction at Tübingen University followed a procedure modified from Longin^71^ described by Bocherens *et al*.^73^. Stable isotopic measurements (^13^C, ^15^N) used an elemental analyser NC 2500 connected to a Thermo Quest Delta+XL mass spectrometer. The degree of chemical preservation of collagen is expressed as the atomic ratio of C_coll_:N_coll_, whose acceptable range of variation is 2.9–3.6^74^, while the nitrogen content (N_coll_) should be above 5%^75^. The carbon content of the extracted collagen ranges between 29.5 and 47.1% and nitrogen content between 10.1 and 17.0% (Supplementary Table S3), both of which fall in the range of fresh collagen^76^. The C_coll_:N_coll_ atomic ratios span a narrow range (3.2–3.4), indicating exceptionally well-preserved collagen for all bone specimens (see Supplementary Note S6 for discussion of tooth 2878–2D). Subsequently, the collagen was sent to the CIO for ^14^C dating by AMS^77^.

### Genetic analyses

Ten specimens were sampled (Table 1). DNA was extracted^78^ from bone powder and converted to double-indexed genetic libraries^79^,^80^. Mitochondrial DNA (mtDNA) was enriched using a bait capture technique^81^ and sequenced on a next generation sequencing platform (Illumina HiSeq). After quality filtering and merging paired-end reads^82^, a modified version of the BWA mapper and the SAMtools package in combination with a custom iterative mapping assembler^30^,^83^,^84^,^85^ were used to align reads to a reference Neandertal mtDNA sequence (Supplementary Table S7). Reads from the three low coverage specimens were also aligned to the modern human mtDNA reference sequence (rCRS) in order to exclude reference biases (Supplementary Table S8). The newly reconstructed complete or almost complete (i.e. at least 98% complete) mitochondrial genomes were compared with 63 other hominin mtDNA sequences in gene trees (Fig. 2 and Supplementary Fig. S4) to assess their phylogenetic placements and intergroup genetic relationships (Supplementary Note S7). The authenticity of the obtained mitochondrial sequences as endogenous ancient DNA was verified by analysing typical ancient DNA damage patterns (ref. 86 and Supplementary Fig. S17) as well as estimating the percentage of modern human DNA contamination (ref. 30 and Supplementary Table S7). Finally, damaged DNA molecules indicating an ancient origin were filtered^34^ and used to build new mtDNA consensus sequences. These were co-analysed with the same 63 mtDNAs in order to validate the assigned phylogenetic placement (Supplementary Figs S18 and S19).

## Additional Information

**Accession codes:** The seven Neandertal mtDNA sequences reported in this article were deposited in GenBank and are available under accession numbers KX198082-KX198088.

**How to cite this article**: Rougier, H. *et al*. Neandertal cannibalism and Neandertal bones used as tools in Northern Europe. *Sci. Rep.* **6**, 29005; doi: 10.1038/srep29005 (2016).

## Supplementary Material

Supplementary Information

## Figures and Tables

**Table 1:** Sample information and results of the ^14^C and genetic analyses of the Neandertal remains from Goyet.

Specimen		Radiocarbon dating			Genetic analyses	Anthropogenic marks
ID	Description	Lab #	^14^C age (BP)	Calibrated age (calBP) 95% probability		
2878–2D*	Lower lt P2 (mandible 2878–8)	GrA-54028	32,190 +200, −190	36,510–35,630	−	−
C5–1	Lt parietal frag.	−	−	−	Nean	−
Q53–4	Rt humerus diaph. frag. (humerus III)	GrA-54022	39,870 +400, −350	44,330–42,920	−	−
Q55–1	Lt clavicle frag.	GrA-54257	37,860 +350, −310	42,650–41,700	−	C
Q55–4	Rt tibia diaph. frag. (tibia IV)	−	−	−	Nean	C + N + P + R
Q56–1	Rt femur diaph. frag. (femur I)	GrA-46170	38,440 +340, −300	43,000–42,080	1	C + N + P
Q57–1	Lt tibia diaph. frag. (tibia II)	GrA-46173	41,200 +500, −410	45,630–43,910	2	C + N
Q57–2	Rt femur diaph. frag. (femur II)	GrA-54024	36,590 +300, −270	41,800–40,620	2	C + N + P
Q57–3	Rt tibia diaph. frag. (tibia VI)	GrA-60019	38,260 +350, −310	42,900–41,960	2	C + N
Q119–2	Lt rib 7? frag.	−	−	−	Nean	−
Q305–4	Lt tibia diaph. frag. (tibia I)	GrA-46176	40,690 +480, −400	45,150–43,430	3	C + N
Q305–7	Rt tibia diaph. frag. (tibia III)	−	−	−	1	C + N + P + R
Q374a–1	Rt tibia diaph. frag. (tibia V)	−	−	−	1	C + N + P + R
Q376–1	Hand prox. phalanx 2-4	GrA-46178	39,140 +390, −340	43,650–42,440	−	−
Q376–20	Rt humerus diaph. frag. (humerus II)	GrA-60018	37,250 +320, −280	42,240–41,290	−	C + N?

^*^This specimen may have been varnished resulting in a young age (Supplementary Note S6). For the calibration of the ^14^C ages, see Supplementary Note S6. Genetic analyses: 1–3 represent three distinct Neandertal mtDNA lineages, Nean: Neandertal status confirmed; Anthropogenic modifications: C: cutmarks, N: percussion notches, P: percussion pits, R: retoucher traces. All of the specimens are part of the RBINS collections and were excavated by E. Dupont in 1868.

**Table 2:** Numbers and proportions of Neandertal, horse, reindeer and carnivore remains bearing anthropogenic modifications and toothmarks in the Goyet assemblage.

	Nean dertal	Horse	Reindeer	Carnivore
*NISP Observed*	*96*	*442*	*287*	*89*
NISP Cutmarks	31 (32%)	85 (19%)	126 (44%)	3 (3%)
NISP Percussion Notches	20 (21%)	107 (24%)	151 (53%)	0
NISP Percussion Pits	10 (10%)	6 (1%)	1 (0.3%)	0
NISP Retoucher Traces	5 (5%)	22 (5%)	58 (20%)	0
NISP Toothmarks	1 (1%)	27 (6%)	4 (1%)	17 (19%)

Carnivores include bear (*Ursus spelaeus* or *Ursus arctos*), fox (*Vulpes vulpes* or *Vulpes lagopus*), a large canid (*Canis sp.*), hyaena (*Crocuta crocuta spelaea*), and badger (*Meles meles*). The observed faunal specimens were identified among a sample of Dupont’s collection from FBL 2 and 3 (Supplementary Table S5). Note that the high percentage of retouchers made on reindeer bones is most likely related to the under-representation of fragments less than 55 mm long in our sample.

**Table 3:** Description of the Neandertal bone retouchers from Goyet using the criteria of Mallye *et al*. 40 and Daujeard *et al*. 41.

		Femur III anterior area	Femur III medial area	Tibia III posterior area	Tibia IV posterior area	Tibia V medial area
Area	Length (mm)	14.6	19.2	11.4	17.0	20.9
	Width (mm)	4.4	7.5	9.0	5.8	13.2
	Preparatory scraping	no	no	no	no	no
	Morphology (if concentrated and superposed traces)	−	hatched	−	−	hatched
Marks	Orientation (to the long axis of the fragment)	oblique	transverse	transverse	transverse	transverse, slightly oblique
	Position	centered	centered?	centered	centered	centered
	Concentration	dispersed	concentrated and superposed	dispersed	dispersed	concentrated and superposed
	Morphology	rectilinear - smooth	rectilinear - rough	rectilinear - smooth	rectilinear - smooth and rough	rectilinear - smooth
