# Supplementary material for: Neandertal cannibalism and Neandertal bones used as tools in Northern Europe
Source: Sci Rep. 2016 Jul 6;6:29005. doi: 10.1038/srep29005 (PMC4933918; doi:10.1038/srep29005)
Supplement: Supplementary Information [file srep29005-s1.pdf]

# Neandertal cannibalism and Neandertal bones used as tools in Northern Europe – Supplementary Information

Hélène Rougier, Isabelle Crevecoeur, Cédric Beauval, Cosimo Posth, Damien Flas, Christoph Wißing, Anja Furtwängler, Mietje Germonpré, Asier Gómez-Olivencia, Patrick Semal, Johannes van der Plicht, Hervé Bocherens & Johannes Krause

## Table of Contents

**Supplementary Figures ........ 3**

Supplementary Fig. S1. Map of Northern Europe centred on Belgium (top) and detail of the central area (bottom) showing the location of Goyet and other sites above 50° north that yielded late Neandertal remains. ........ 3
Supplementary Fig. S2. The 21 drawers of fragmentary, “indeterminate” fauna from Dupont’s excavations at the Troisième caverne of Goyet that were systematically sorted in order to identify any overlooked human remains. ........ 4
Supplementary Fig. S3. Left and right tibia pieces and lower left lateral incisor used to estimate the Neandertal MNI for Goyet. ........ 5
Supplementary Fig. S4. Maximum likelihood tree for the seven analysed Goyet samples that produced complete or almost complete mitochondrial genomes compared to 63 published modern human, Neandertal and Denisovan mtDNAs ........ 6
Supplementary Fig. S5. Tibia I, the most complete refit piece (8 refits) from the Neandertal assemblage. ..7
Supplementary Fig. S6. The Neandertal hand phalanges Q376-1, 2878-37, 2878-38, and 2878-39 (from left to right) in dorsal (a) and palmar (b) view and the Neandertal rib Q376-26 in posterior view (c) showing traces of peeling (d). ........ 8
Supplementary Fig. S7. Number of Neandertal bones (after refitting) with cutmarks (left), percussion notches (centre) and retoucher traces (right). ........ 9
Supplementary Fig. S8. Drawings of the Goyet Neandertal remains bearing anthropogenic modifications. ........ 10
Supplementary Fig. S9. Femur III in medial (a) and anterior (b) view and details of the two areas of the bone used as retouchers. ........ 11
Supplementary Fig. S10. Tibia III in posterior view (a) and details of the area of the bone used as a retoucher (b and c). ........ 12
Supplementary Fig. S11. Tibia IV in posterior view (a) and details of the area of the bone used as a retoucher (b and c). ........ 13
Supplementary Fig. S12. Tibia V in medial view (a) and details of the area of the bone used as a retoucher (b and c)……. ........ 14
Supplementary Fig. S13. Skeletal representation of the Neandertal (left), horse (top right) and reindeer (bottom right) remains. ........ 15
Supplementary Fig. S14. Cutmarks on horse (top left), reindeer (top right) and Neandertal (bottom) bones from Goyet……. ........ 16
Supplementary Fig. S15. Percussion notches on horse (top left), reindeer (top right) and Neandertal bones (bottom) from Goyet. ........ 17
Supplementary Fig. S16. Retoucher traces on horse (top left), reindeer (top right) and Neandertal (bottom) bones from Goyet. ........ 18
Supplementary Fig. S17. Damage plots for all of the Goyet Neandertal samples before postmortem damage (PMD) filtering. ........ 19
Supplementary Fig. S18. Maximum parsimony tree of the seven analysed Goyet Neandertal mitochondrial genomes after selection of damaged reads (PMD filtering) compared to 63 published modern human, Neandertal and Denisovan mtDNAs. ........ 20
Supplementary Fig. S19. Maximum likelihood tree of the seven analysed Goyet Neandertal mitochondrial genomes after selection of damaged reads (PMD filtering) compared to 63 published modern human, Neandertal and Denisovan mtDNAs. ........ 21

**Supplementary Tables ........ 22**

Supplementary Table S1. Human remains from the Troisième caverne of Goyet identified as Neandertal with indication of the “fauna-bearing level” (FBL) and analyses performed. ........ 22

Supplementary Table S2. Neandertal bones (after refitting) from the Troisième caverne of Goyet with indication of anthropogenic modifications. ....24
Supplementary Table S3. Sample information and results of the elemental chemical analyses of the Neandertal remains from Goyet. ....26
Supplementary Table S4. Minimum number of elements (MNE) and minimum number of individuals (MNI) represented by the Goyet Neandertal bone assemblage along with the percentage representation (PR) of the elements. ....27
Supplementary Table S5. Faunal sample from Dupont's excavations at the Troisième caverne of Goyet identified during the present study with indication of the storage drawers and “fauna-bearing levels” (FBL)…… ....28
Supplementary Table S6. Comparison of the horse, reindeer and Neandertal skeletal representation at Goyet………… ....29
Supplementary Table S7. Palaeogenetic results of the mtDNA reads for the 10 Goyet Neandertal specimens analysed before and after postmortem damage (PMD) filtering. ....30
Supplementary Table S8. Palaeogenetic results of the mtDNA reads for the three low coverage specimens mapped against modern human and Neandertal mitochondrial reference sequences before postmortem damage filtering. ....31

**Supplementary Notes .... 32**

Supplementary Note S1. The Troisième caverne of Goyet and its regional context ....32
Supplementary Note S2. Assessment of the Goyet Middle Palaeolithic material and overview of the Late Mousterian from the Mosan Basin ....33
Supplementary Note S3. Reassessment of the Goyet collections ....33
Supplementary Note S4. Labelling and provenience of the Goyet Neandertal remains ....34
Supplementary Note S5. The Neandertal assemblage from the Troisième caverne ....35
Supplementary Note S6. Radiocarbon dating of the Goyet Neandertals ....37
Supplementary Note S7. Palaeogenetic analyses of the Goyet Neandertals ....37

**Supplementary Information References .... 40**

## Supplementary Figures

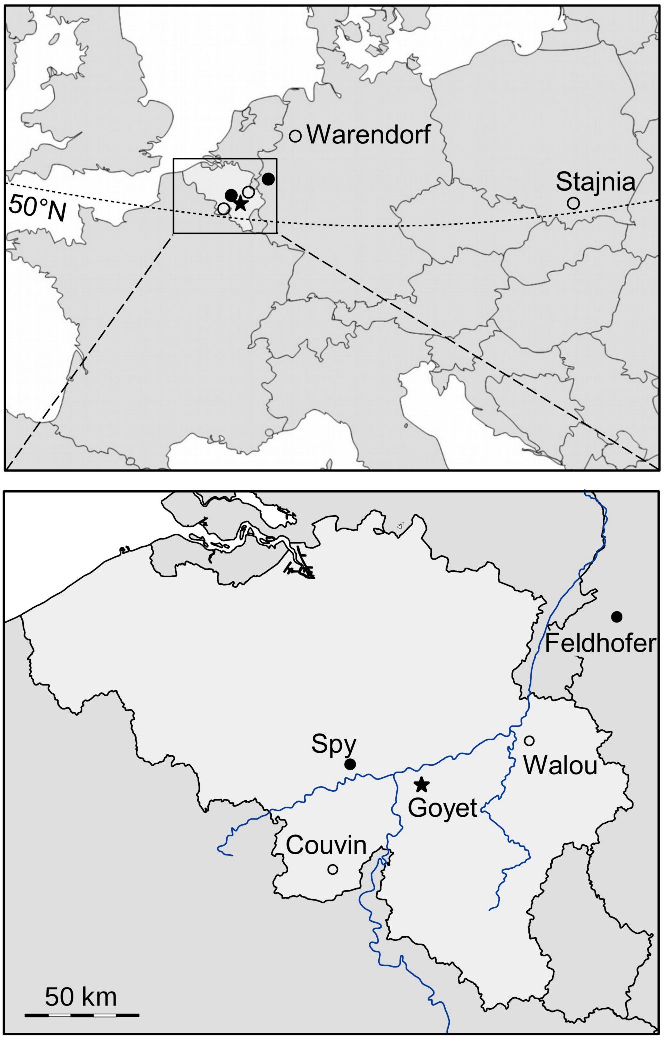


**Supplementary Fig. S1. Map of Northern Europe centred on Belgium (top) and detail of the central area (bottom) showing the location of Goyet and other sites above 50° north that yielded late Neandertal remains.**

Black and white circles indicate, respectively, sites with directly- and indirectly-dated Neandertal remains. Map created using LibreOffice 5.0 Draw and exported in PDF 600 DPI (http://www.libreoffice.org/); Tiff file generated from the PDF using GIMP 2.8 (https://www.gimp.org).

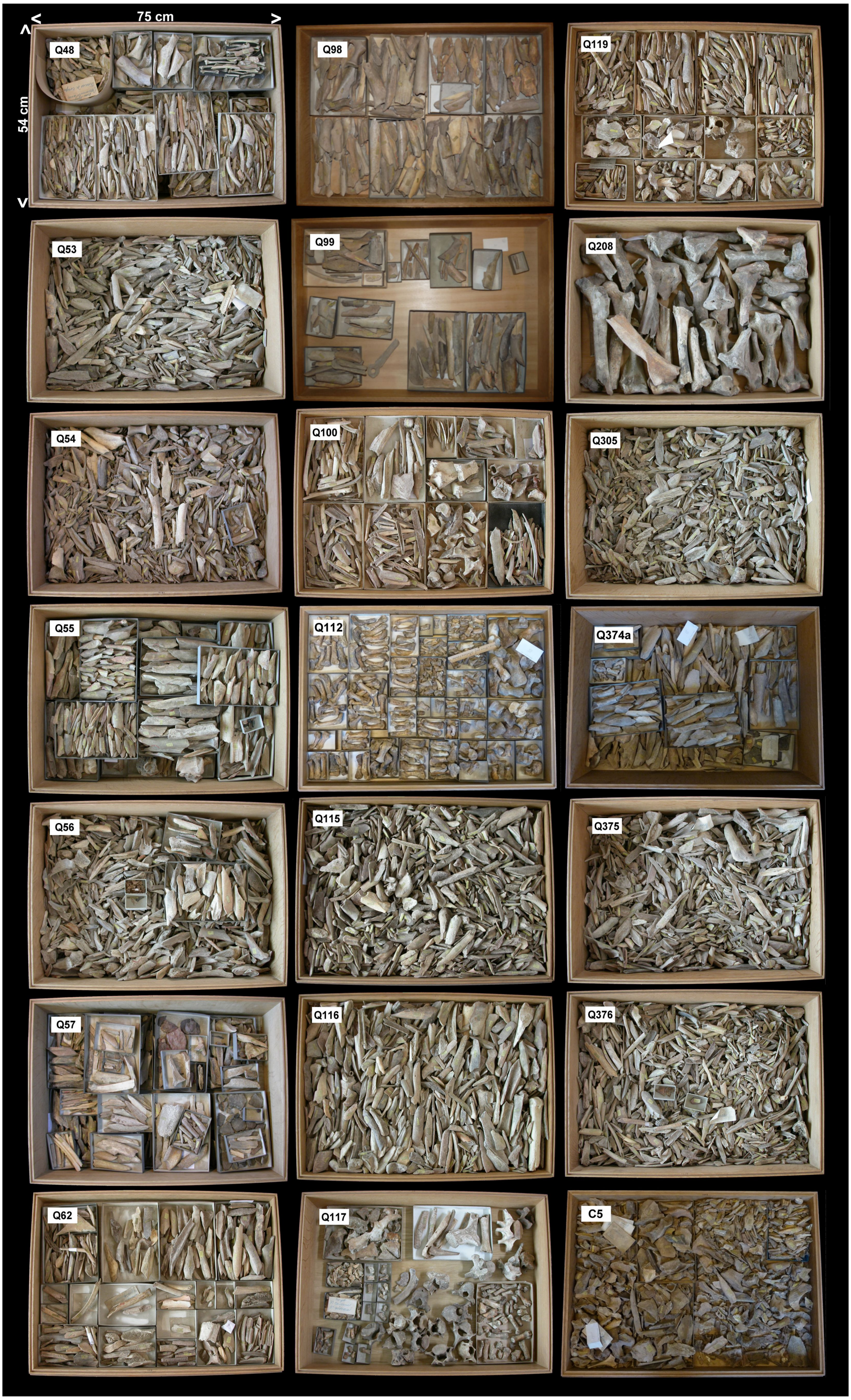


**Supplementary Fig. S2. The 21 drawers of fragmentary, "indeterminate" fauna from Dupont's excavations at the Troisième caverne of Goyet that were systematically sorted in order to identify any overlooked human remains.**

The comparative taphonomic analysis of the Goyet Neandertal remains was conducted on the faunal remains identified in drawers Q53, Q55, Q375, and Q376 (see Supplementary Table S5).

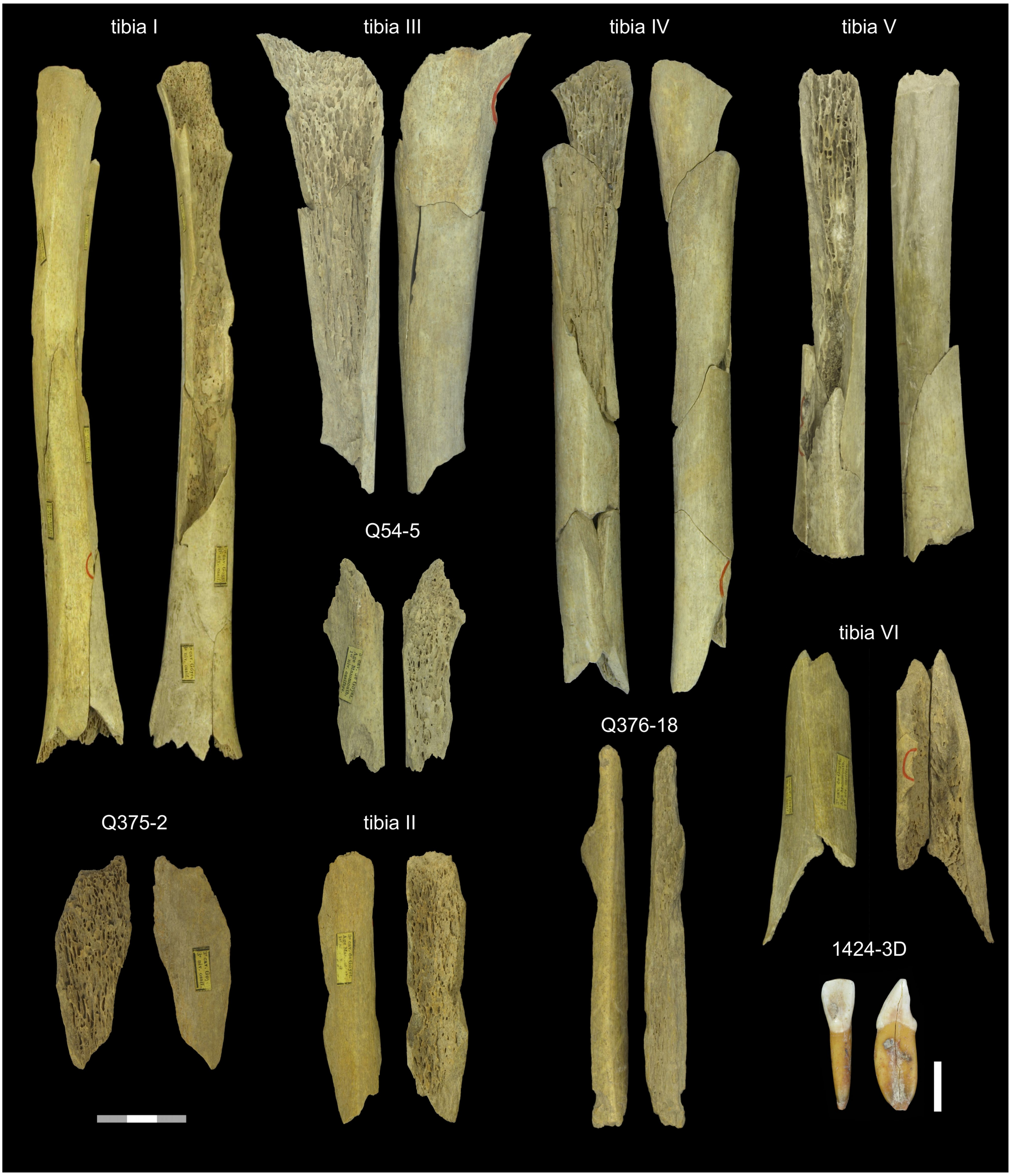


**Supplementary Fig. S3. Left and right tibia pieces and lower left lateral incisor used to estimate the Neandertal MNI for Goyet.**

Anterior (left) and posterior (right) view of each tibia piece; lingual (left) and distal (right) view of the lower left lateral incisor. Tibias I and II and Q375-2 are from the left side; all of the other tibia pieces are from the right side. Scale = 3 cm for the bone pieces and 1 cm for the tooth.

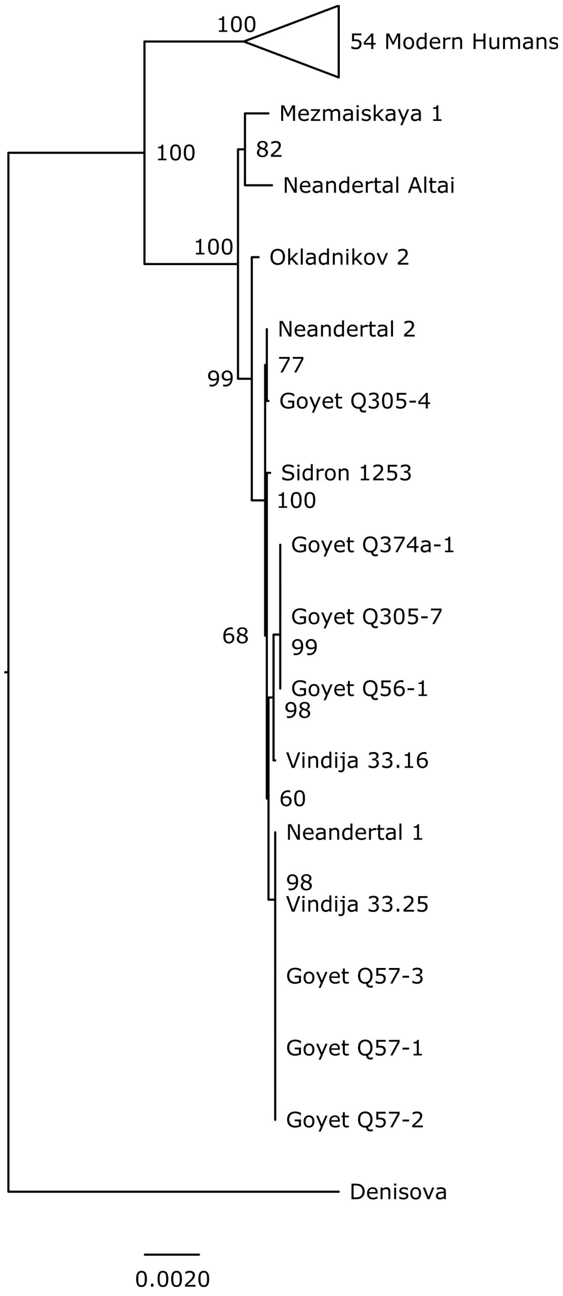


**Supplementary Fig. S4. Maximum likelihood tree for the seven analysed Goyet samples that produced complete or almost complete mitochondrial genomes compared to 63 published modern human, Neandertal and Denisovan mtDNAs.**

Numbers at the main branch nodes represent bootstrap values after 1,000 iterations.

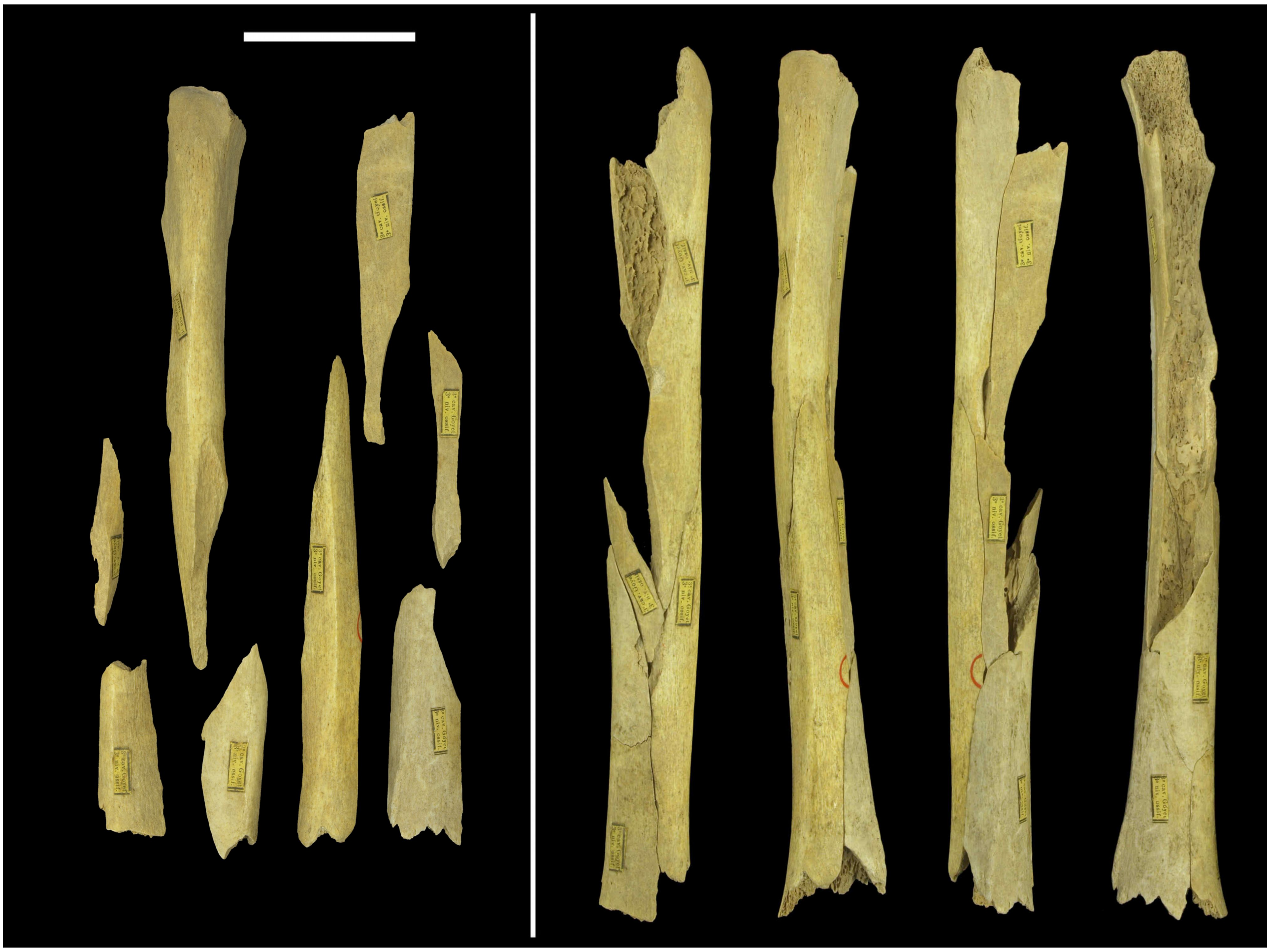

**Supplementary Fig. S5. Tibia I, the most complete refit piece (8 refits) from the Neandertal assemblage.**

Left: individual specimens; right: refit piece (from left to right: in medial, anterior, lateral and posterior views). Note that all of the specimens were found mixed with fauna from E. Dupont excavations and a small yellow label indicating their stratigraphic origin was glued to each at the beginning of the 20th century; red traces were also drawn on likely faunal fragments to delimit impact notches and retoucher areas. Scale = 5 cm.

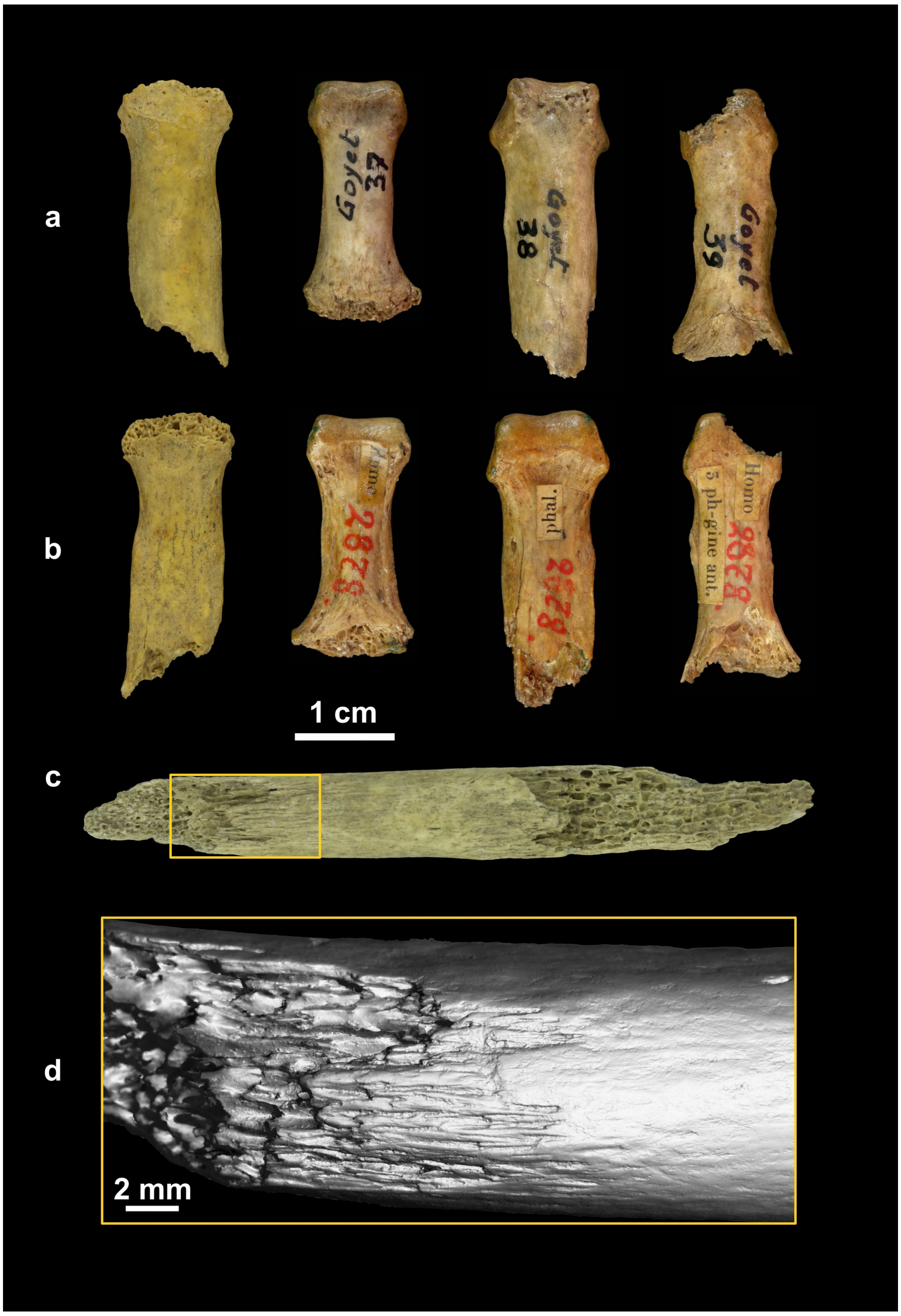


**Supplementary Fig. S6. The Neandertal hand phalanges Q376-1, 2878-37, 2878-38, and 2878-39 (from left to right) in dorsal (a) and palmar (b) view and the Neandertal rib Q376-26 in posterior view (c) showing traces of peeling (d).**

Image d was obtained using a minidome (see Methods).

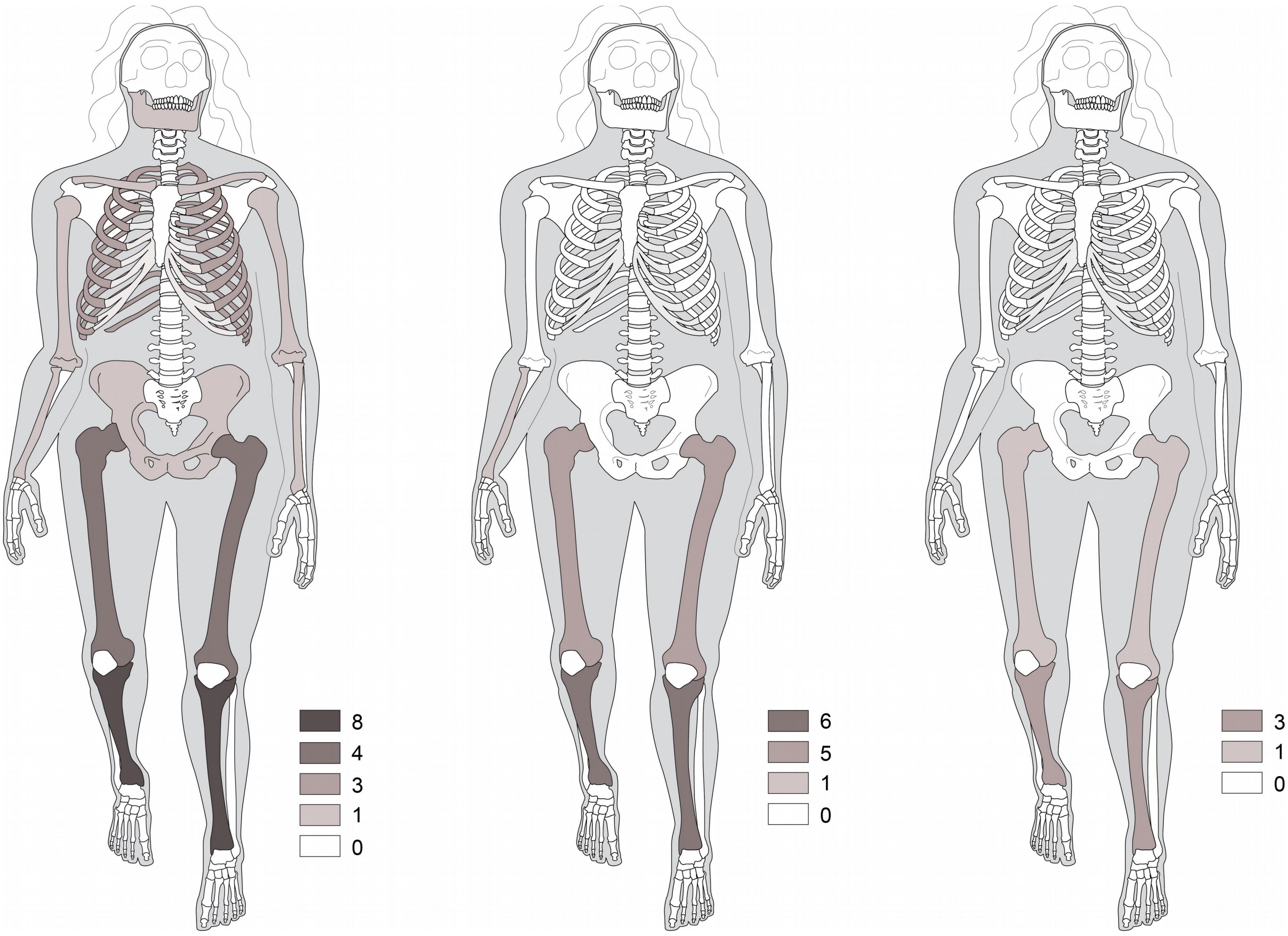


**Supplementary Fig. S7. Number of Neandertal bones (after refitting) with cutmarks (left), percussion notches (centre) and retoucher traces (right).**

Neandertal diagram modified from http://archeozoo.org/archeozootheque/index/category/102-hominides_langen_hominid_lang_langes_hominidos_lang_ (diagram by C.B–© 2013 ArcheoZoo.org, after ref. 43) using Adobe Illustrator CS4 v. 14.0.0.

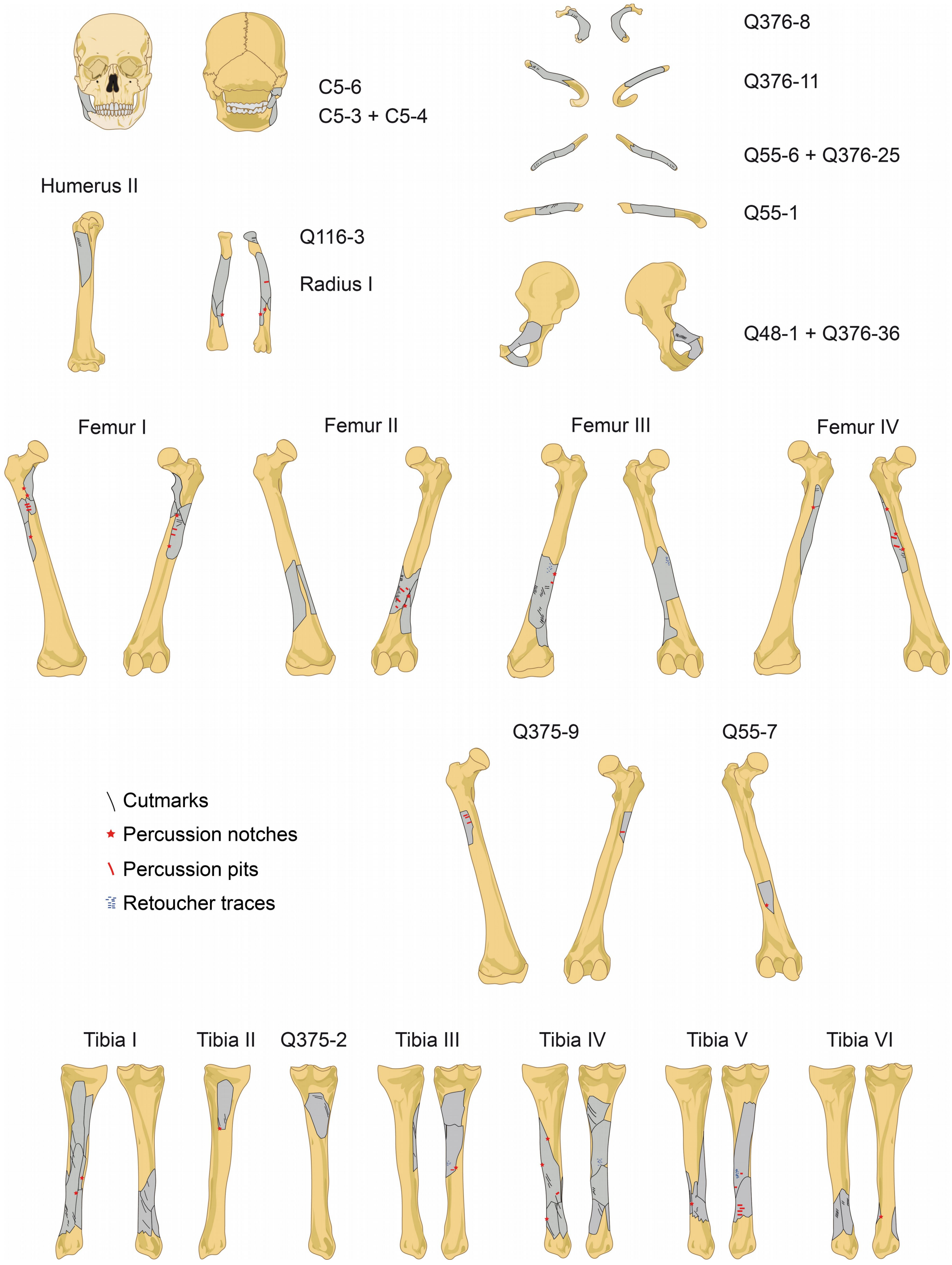


**Supplementary Fig. S8. Drawings of the Goyet Neandertal remains bearing anthropogenic modifications.**

Bone identifications are given in Supplementary Table S2. Grey areas indicate preserved bone portions. Sufficiently preserved elements are shown in both anterior (left) and posterior (right) views. Bone diagrams modified from https://en.wikipedia.org/wiki/File:Human_skeleton_front_en.svg and https://en.wikipedia.org/wiki/File:Human_skeleton_back_en.svg using Adobe Illustrator CS4 v. 14.0.0.

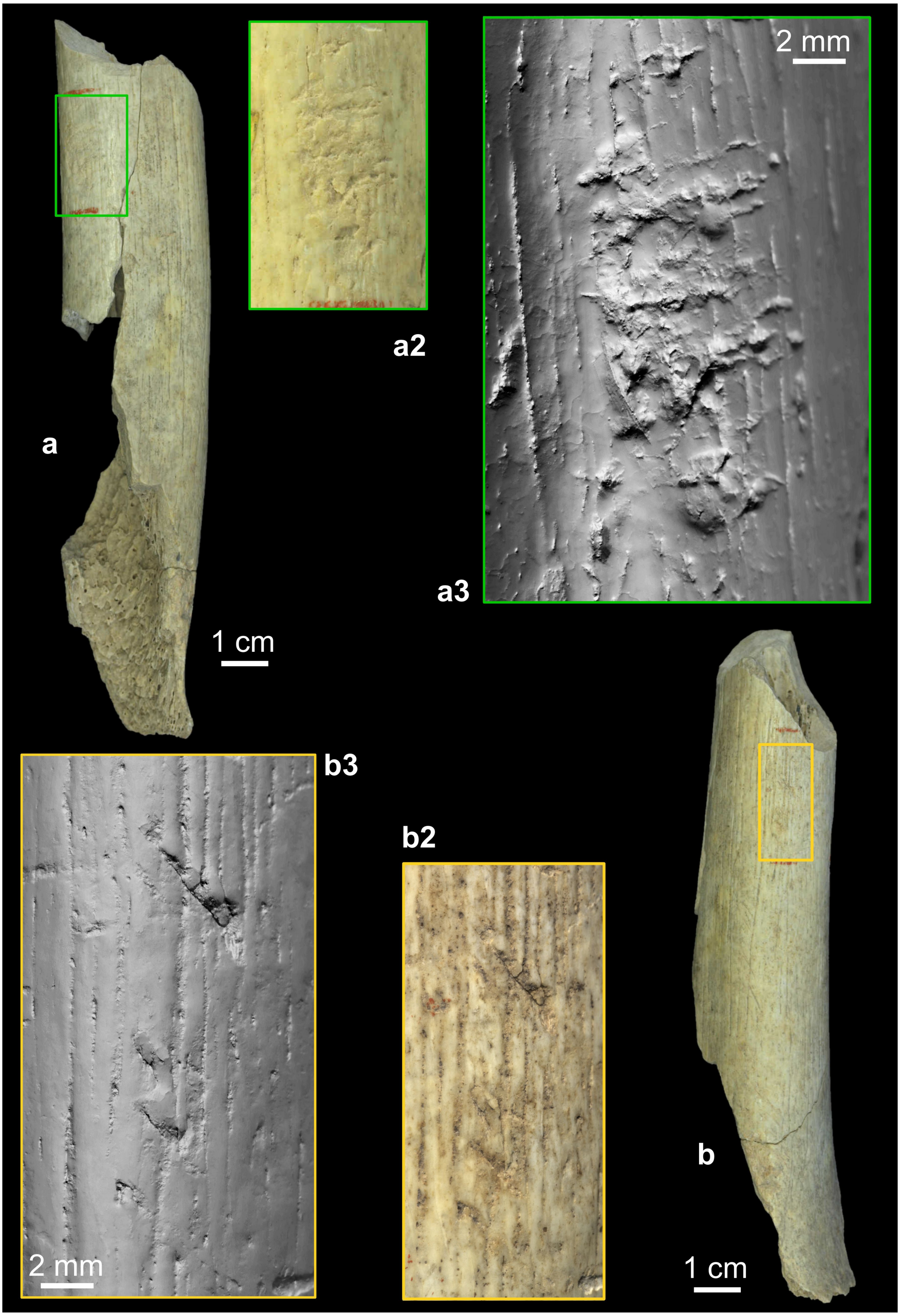


**Supplementary Fig. S9. Femur III in medial (a) and anterior (b) view and details of the two areas of the bone used as retouchers.**

a2 and b2: close-up photos of the areas showing retouching marks; a3 and b3: images of the areas showing retouching marks obtained using a minidome, see Methods.

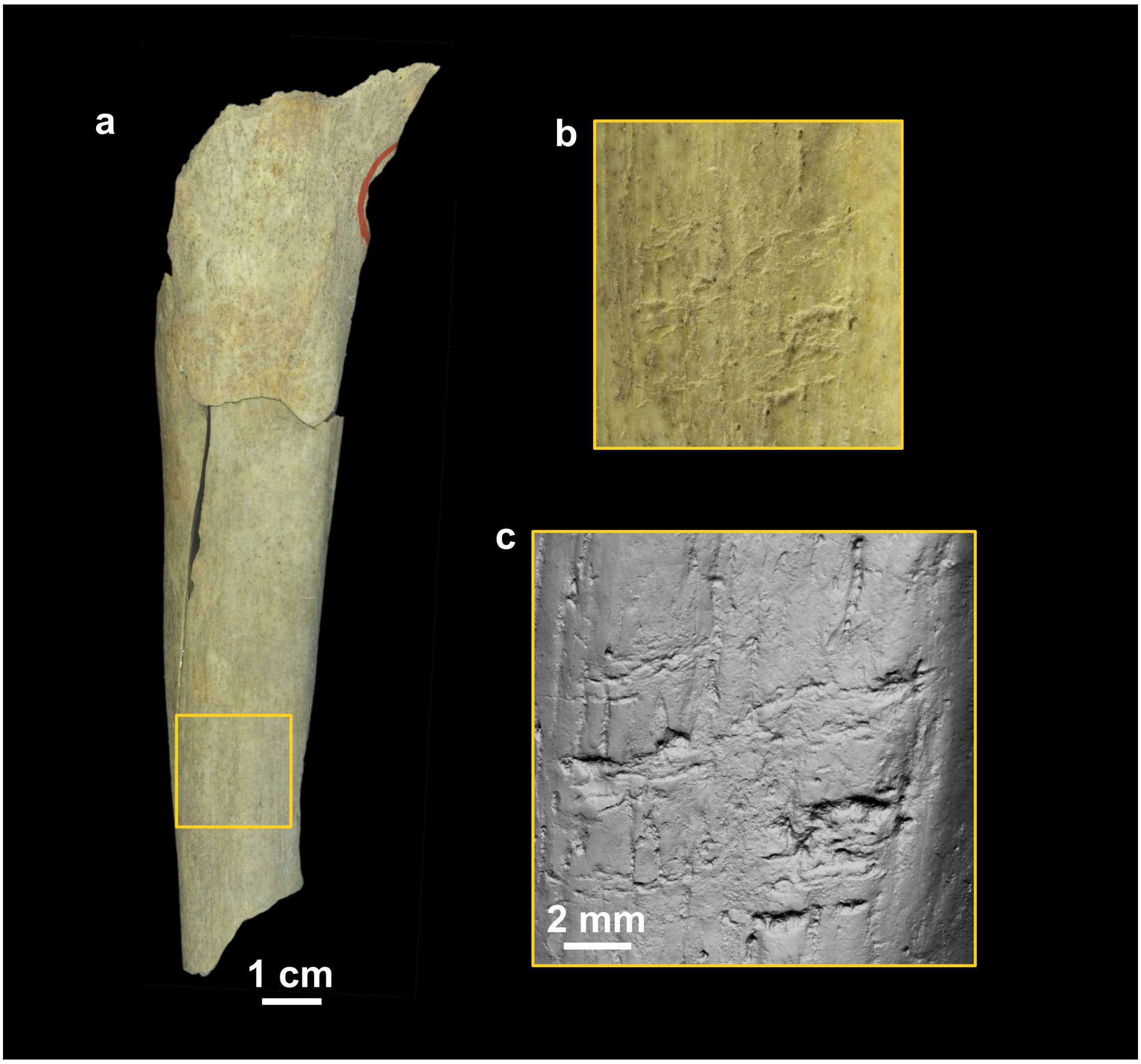


**Supplementary Fig. S10. Tibia III in posterior view (a) and details of the area of the bone used as a retoucher (b and c).**

b: close-up photo of the area showing retouching marks; c: image of the area showing retouching marks obtained using a minidome, see Methods.

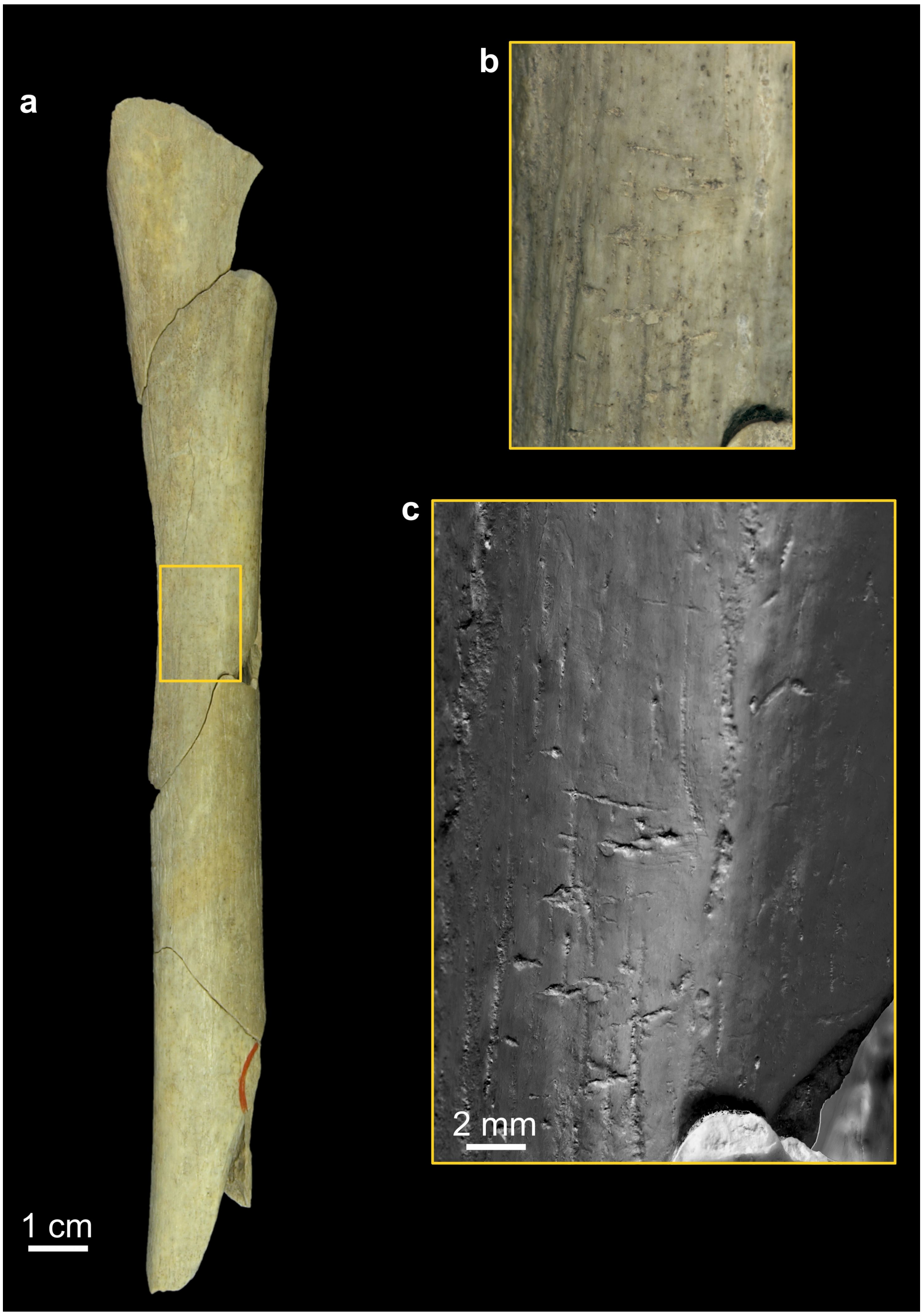


**Supplementary Fig. S11. Tibia IV in posterior view (a) and details of the area of the bone used as a retoucher (b and c).**

b: close-up photo of the area showing retouching marks; c: image of the area showing retouching marks obtained using a minidome, see Methods.

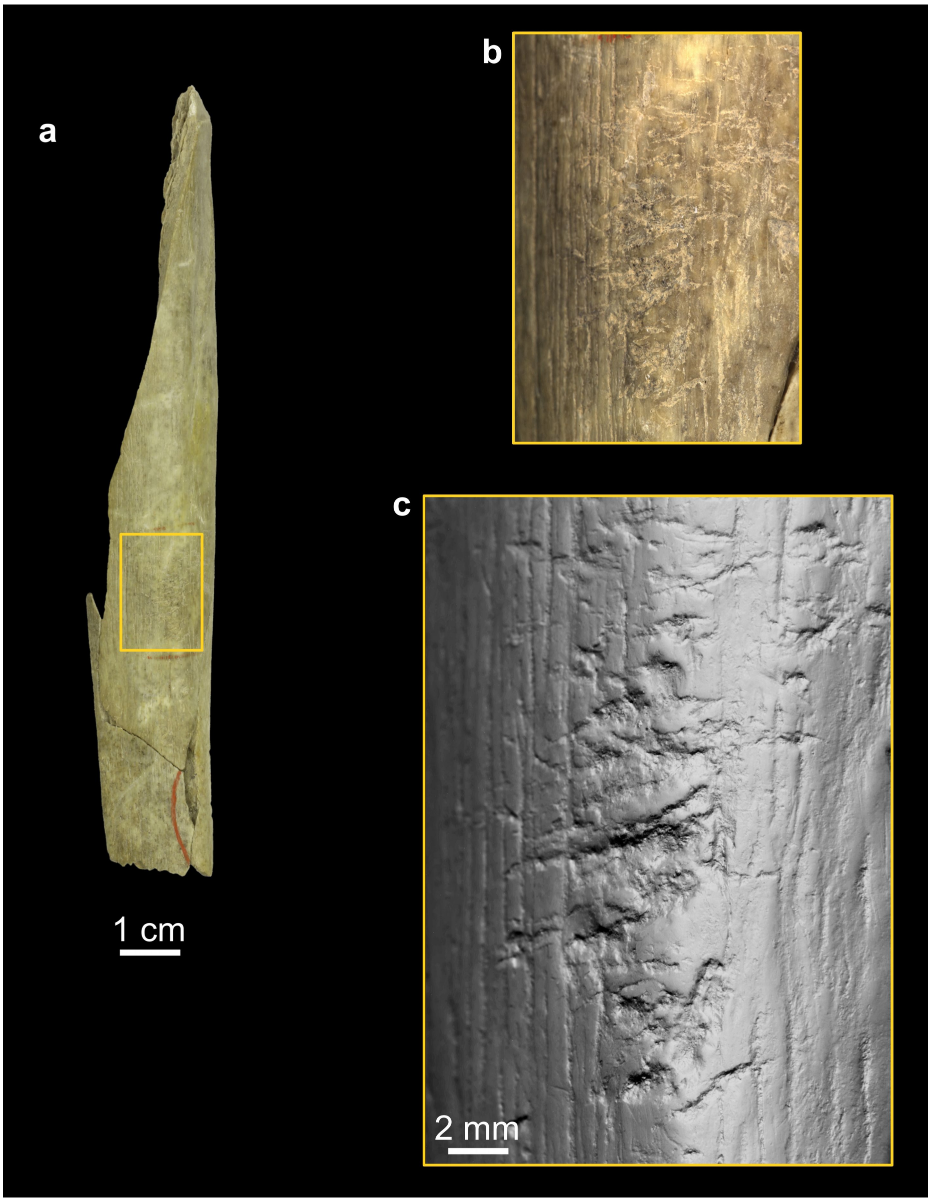


**Supplementary Fig. S12. Tibia V in medial view (a) and details of the area of the bone used as a retoucher (b and c).**

b: close-up photo of the area showing retouching marks; c: image of the area showing retouching marks obtained using a minidome, see Methods.

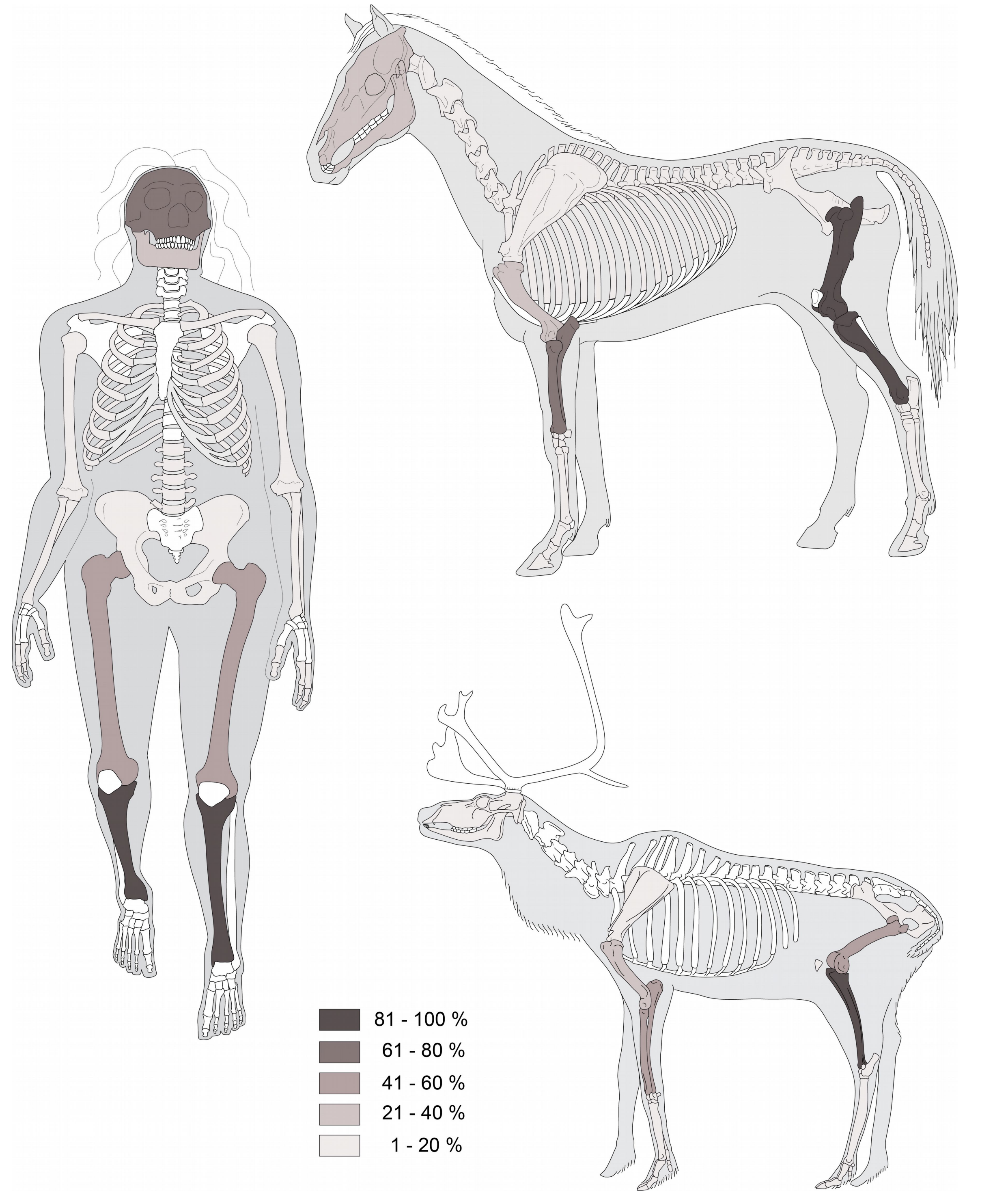


**Supplementary Fig. S13. Skeletal representation of the Neandertal (left), horse (top right) and reindeer (bottom right) remains.**

See Supplementary Table S6 for the values of each species. Neandertal diagram modified from http://archeozoo.org/archeozootheque/index/category/102-hominides_langen_hominid_lang_langes_hominidos_lang_ (diagram by C.B–© 2013 ArcheoZoo.org, after ref. 43), horse diagram from http://archeozoo.org/archeozootheque/picture/2595-equus_caballus/category/85-perissodactyles_langen_odd_toed_ungulate_lang_langes_perisodactilos_lang_ (diagram by M. Coutureau (Inrap), in coll. with V. Forest–© 1996 ArcheoZoo.org, after ref. 44: p. 21), and reindeer diagram from http://archeozoo.org/archeozootheque/picture/2610-rangifer_tarandus/category/92-cervides_langen_cervidae_lang_langes_cervidos_lang_ (diagram by C.B & M. Coutureau (Inrap)–© 2003 ArcheoZoo.org, after ref. 45: p. 182). All diagrams modified using Adobe Illustrator CS4 v. 14.0.0.

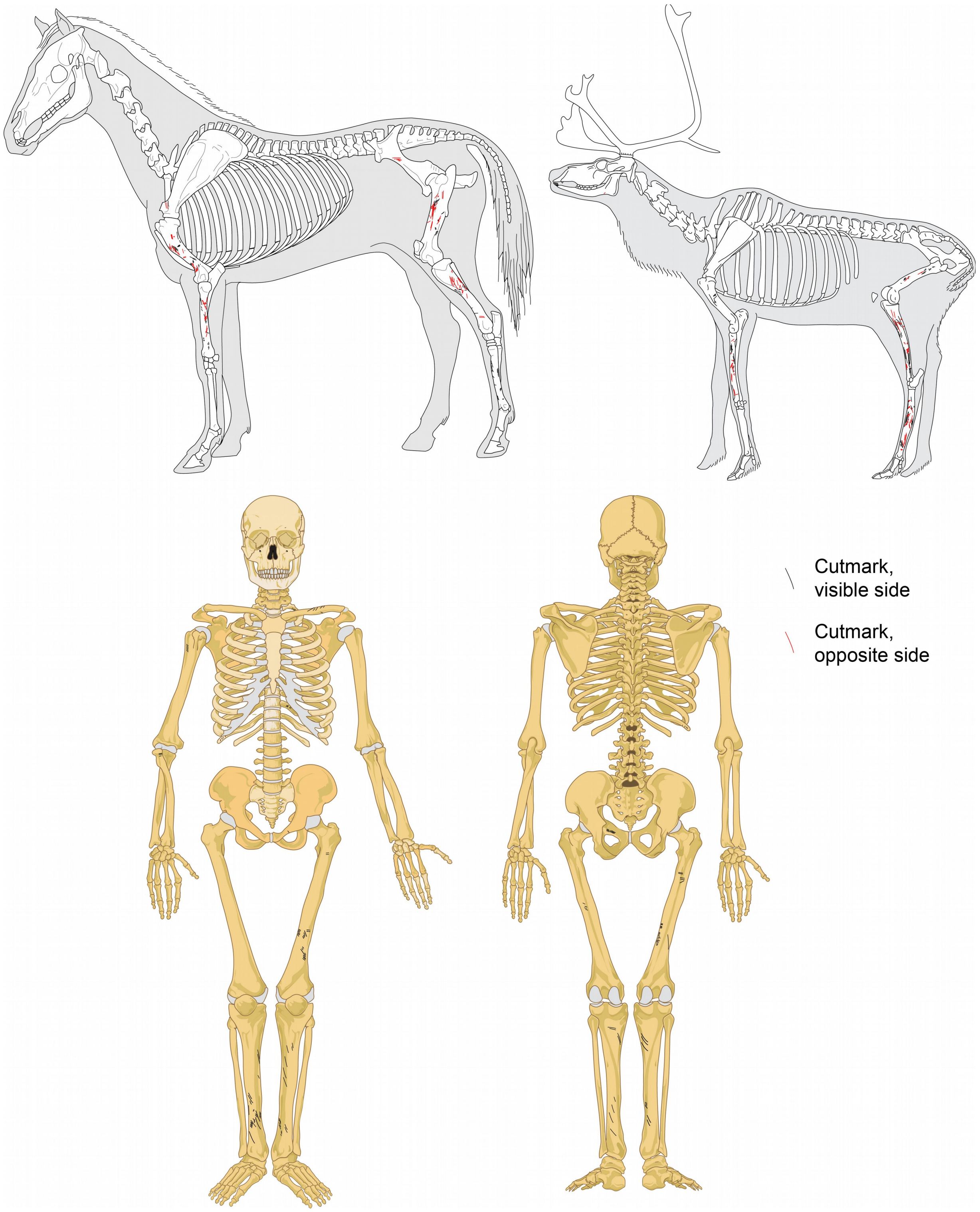


**Supplementary Fig. S14. Cutmarks on horse (top left), reindeer (top right) and Neandertal (bottom) bones from Goyet.**

The faunal specimens were identified among a sample of Dupont's collection from FBL 2 and 3 (horse: N = 442, reindeer: N = 287; Supplementary Table S5). Horse diagram modified from http://archeozoo.org/archeozootheque/picture/2595-equus_caballus/category/85-perissodactyles_langen_odd_toed_ungulate_lang_langes_perisodactilos_lang_ (diagram by M. Coutureau (Inrap), in coll. with V. Forest–© 1996 ArcheoZoo.org, after ref. 44: p. 21), reindeer diagram from http://archeozoo.org/archeozootheque/picture/2610-rangifer_tarandus/category/92-cervides_langen_cervidae_lang_langes_cervidos_lang_ (diagram by C.B & M. Coutureau (Inrap)–© 2003 ArcheoZoo.org, after ref. 45: p. 182), and human skeleton diagrams from https://en.wikipedia.org/wiki/File:Human_skeleton_front_en.svg and https://en.wikipedia.org/wiki/File:Human_skeleton_back_en.svg. All diagrams modified using Adobe Illustrator CS4 v. 14.0.0.

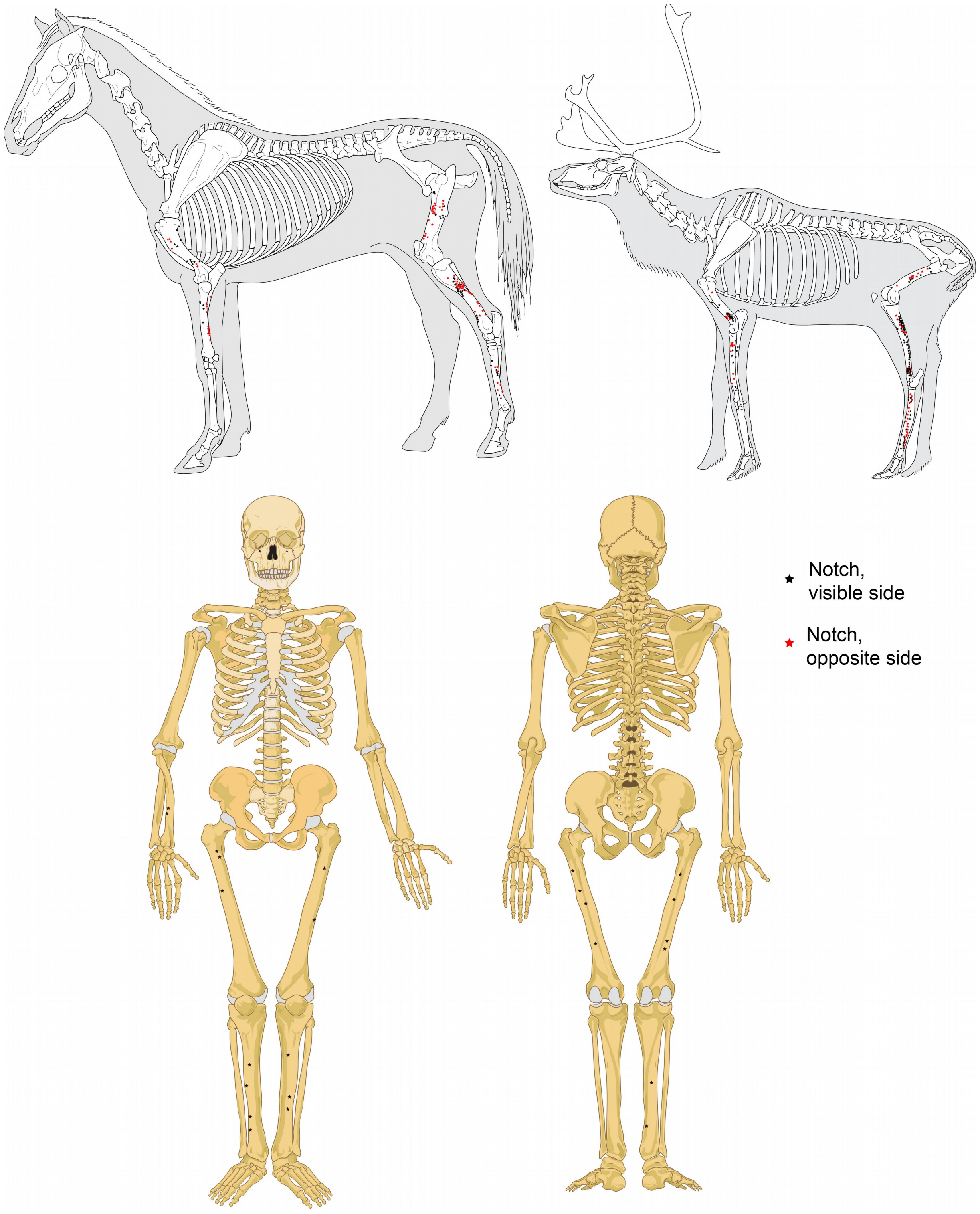


**Supplementary Fig. S15. Percussion notches on horse (top left), reindeer (top right) and Neandertal bones (bottom) from Goyet.**

The faunal specimens were identified among a sample of Dupont's collection from FBL 2 and 3 (horse: N = 442, reindeer: N = 287; Supplementary Table S5). Horse diagram modified from http://archeozoo.org/archeozootheque/picture/2595-equus_caballus/category/85-perissodactyles_langen_odd_toed_ungulate_lang_langes_perisodactilos_lang_ (diagram by M. Coutureau (Inrap), in coll. with V. Forest–© 1996 ArcheoZoo.org, after ref. 44: p. 21), reindeer diagram from http://archeozoo.org/archeozootheque/picture/2610-rangifer_tarandus/category/92-cervides_langen_cervidae_lang_langes_cervidos_lang_ (diagram by C.B & M. Coutureau (Inrap)–© 2003 ArcheoZoo.org, after ref. 45: p. 182), and human skeleton diagrams from https://en.wikipedia.org/wiki/File:Human_skeleton_front_en.svg and https://en.wikipedia.org/wiki/File:Human_skeleton_back_en.svg. All diagrams modified using Adobe Illustrator CS4 v. 14.0.0.

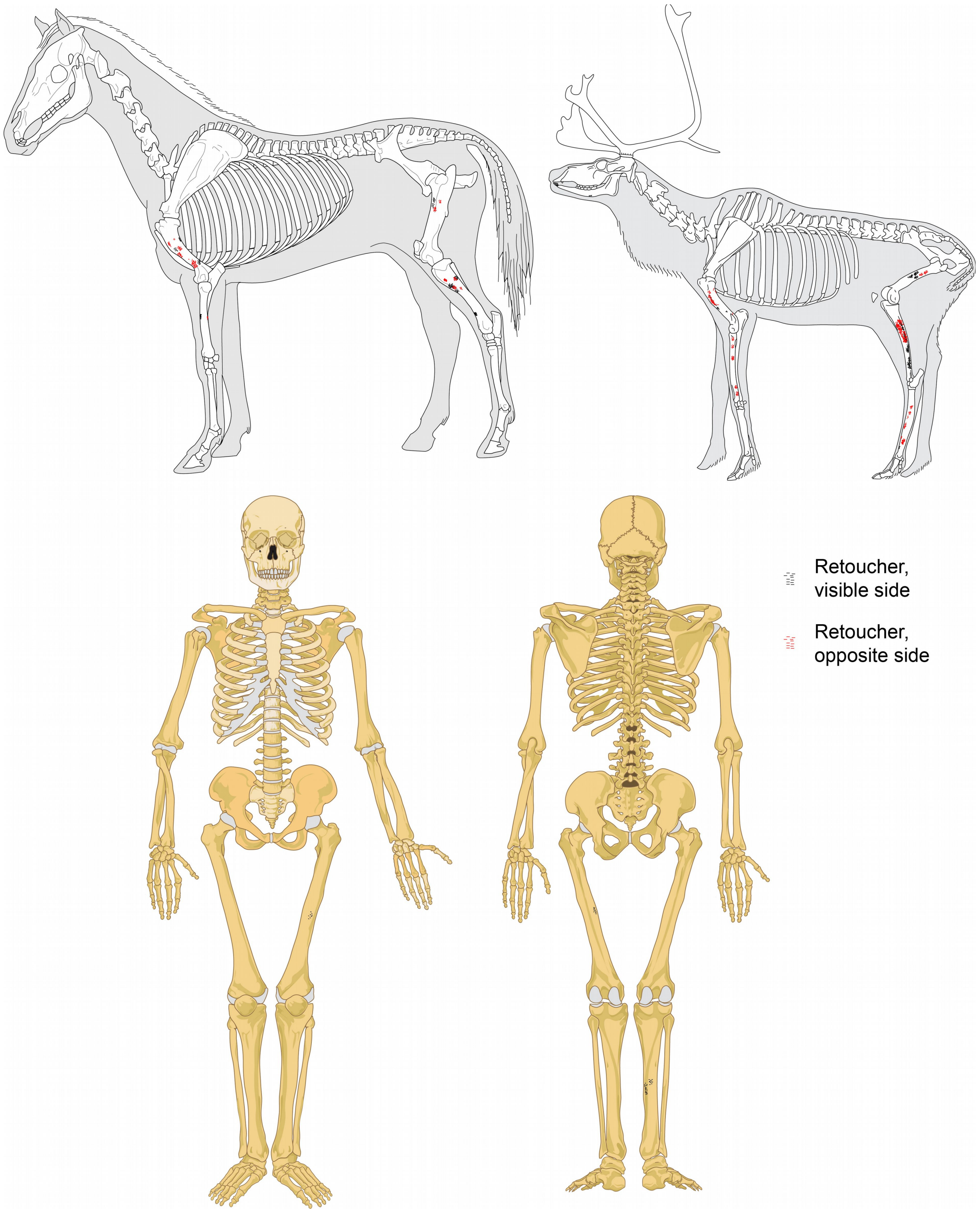


**Supplementary Fig. S16. Retoucher traces on horse (top left), reindeer (top right) and Neandertal (bottom) bones from Goyet.**

The faunal specimens were identified among a sample of Dupont's collection from FBL 2 and 3 (horse: N = 442, reindeer: N = 287; Supplementary Table S5). Horse diagram modified from http://archeozoo.org/archeozootheque/picture/2595-equus_caballus/category/85-perissodactyles_langen_odd_toed_ungulate_lang_langes_perisodactilos_lang_ (diagram by M. Coutureau (Inrap), in coll. with V. Forest–© 1996 ArcheoZoo.org, after ref. 44: p. 21), reindeer diagram from http://archeozoo.org/archeozootheque/picture/2610-rangifer_tarandus/category/92-cervides_langen_cervidae_lang_langes_cervidos_lang_ (diagram by C.B & M. Coutureau (Inrap)–© 2003 ArcheoZoo.org, after ref. 45: p. 182), and human skeleton diagrams from https://en.wikipedia.org/wiki/File:Human_skeleton_front_en.svg and https://en.wikipedia.org/wiki/File:Human_skeleton_back_en.svg. All diagrams modified using Adobe Illustrator CS4 v. 14.0.0.

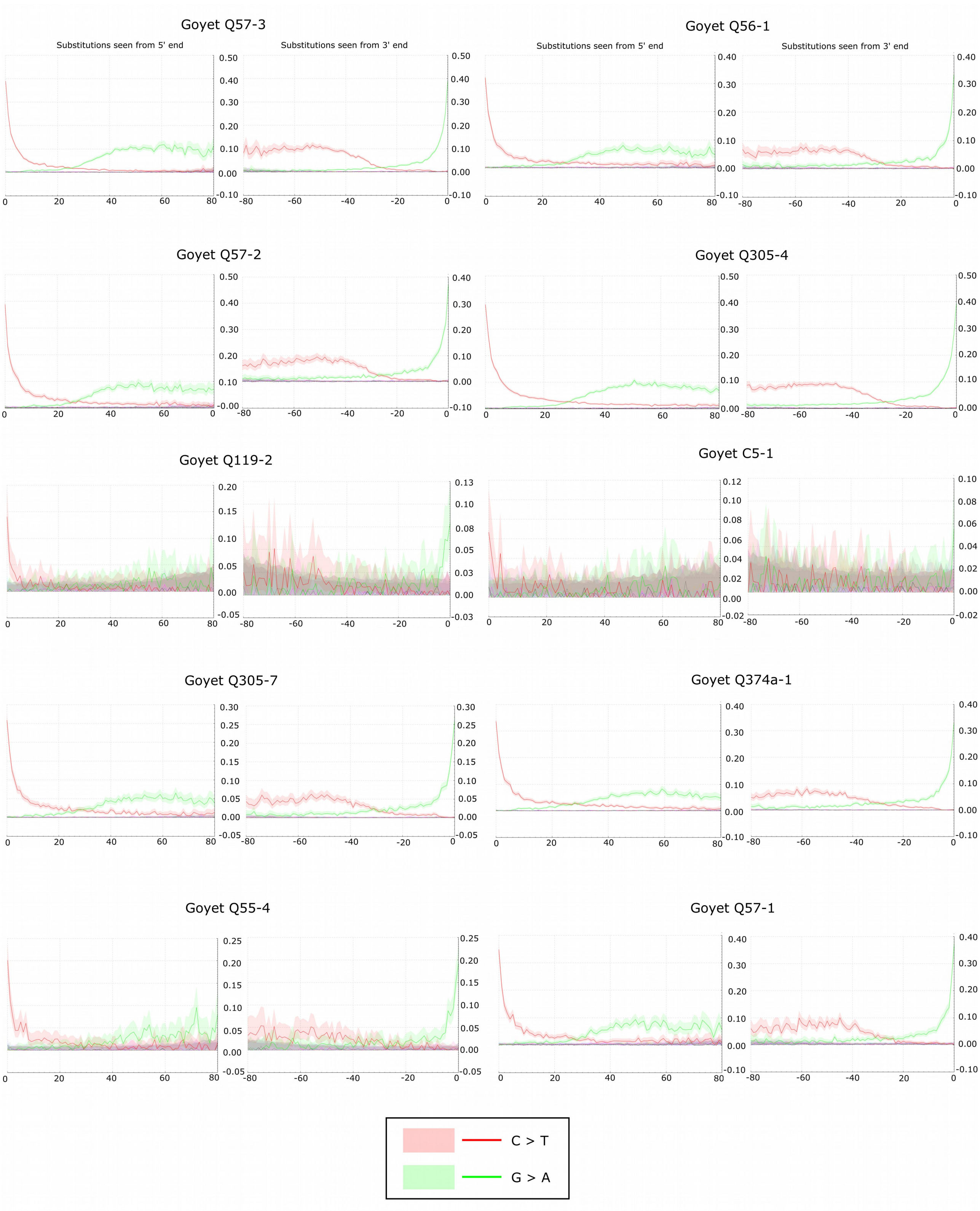


**Supplementary Fig. S17. Damage plots for all of the Goyet Neandertal samples before postmortem damage (PMD) filtering.**

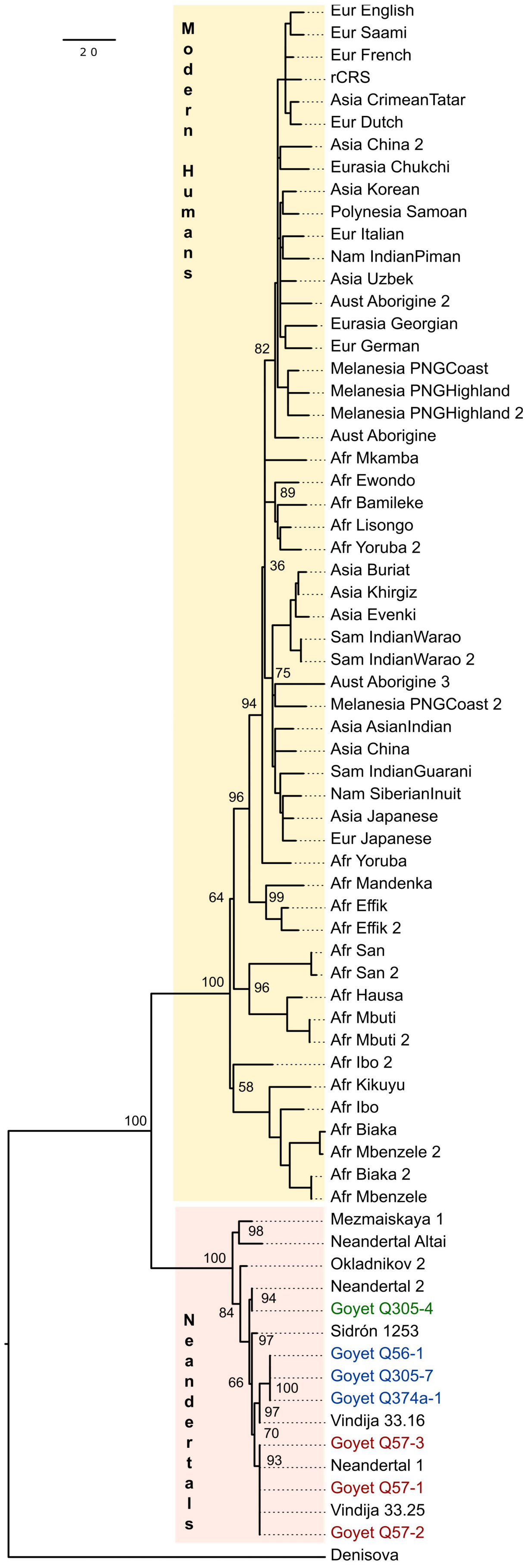


**Supplementary Fig. S18. Maximum parsimony tree of the seven analysed Goyet Neandertal mitochondrial genomes after selection of damaged reads (PMD filtering) compared to 63 published modern human, Neandertal and Denisovan mtDNAs.**

Numbers at the main branch nodes represent bootstrap values after 1,000 iterations.

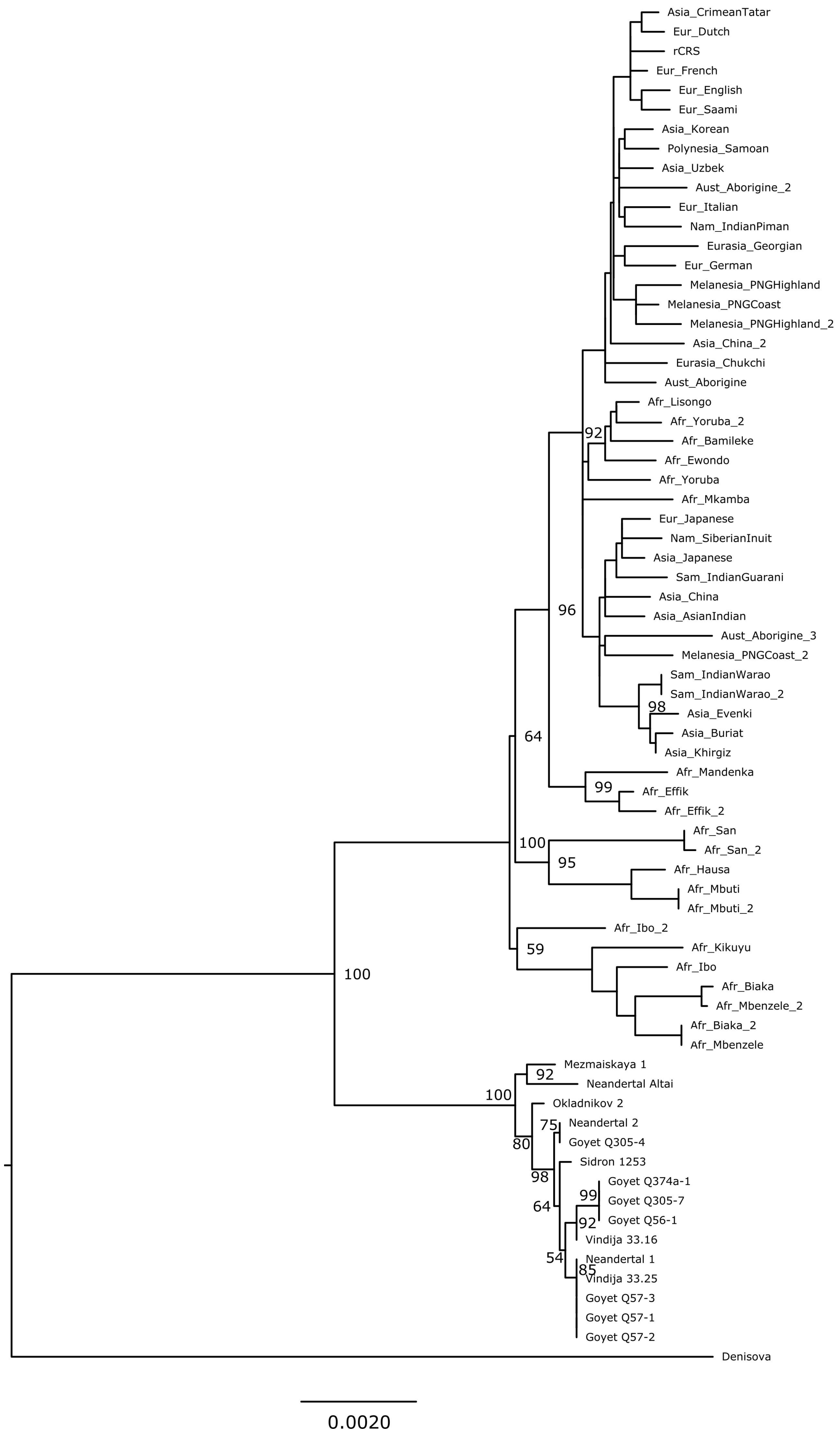


**Supplementary Fig. S19. Maximum likelihood tree of the seven analysed Goyet Neandertal mitochondrial genomes after selection of damaged reads (PMD filtering) compared to 63 published modern human, Neandertal and Denisovan mtDNAs.**

Numbers at the main branch nodes represent bootstrap values after 1,000 iterations.

## Supplementary Tables

### Supplementary Table S1. Human remains from the Troisième caverne of Goyet identified as Neandertal with indication of the "fauna-bearing level" (FBL) and analyses performed.

| Specimen | FBL | Identification | Refits with / on | Analyses | | |
|---|---|---|---|---|---|---|
| | | | | $^{14}C$ | $^{13}C$-$^{15}N$ | DNA |
| 1189-1 | 2 | Left femur diaphysis frag. | Femur III | | | |
| 1424-3D* | / | Lower left I2 (isolated) | | | | |
| 2861-1 | 4? | Right and left maxillae, alveolar and palatine processes | 2878-1D | | | |
| 2861-19D | 4? | Root of upper left I1 | on 2861-1 | | | |
| 2878-1D | 3? | Upper left I2 (isolated) | 2861-1 | | | |
| 2861-20D | 4? | Root of upper left C | on 2861-1 | | | |
| 2861-21D | 4? | Root of upper left P1 | on 2861-1 | | | |
| 2861-22D | 4? | Upper left P2 | on 2861-1 | | | |
| 2861-23D | 4? | Upper left M1 | on 2861-1 | | | |
| 2861-24D | 4? | Upper left M2 | on 2861-1 | | | |
| 2861-25D | 4? | Root of upper right C | on 2861-1 | | | |
| 2861-26D | 4? | Root of upper right P1 | on 2861-1 | Xº | | |
| 2861-27D | 4? | Root of upper right P2 | on 2861-1 | | | |
| 2878-1 | 1 or 3 | Right parietal, postero-superior frag. | | | | |
| 2878-2 | 1 or 3 | Right parietal, anterior frag. | | | | |
| 2878-3 | 1 or 3 | Right and left parietal fragments articulating along the sagittal suture | C5-1 | | | |
| 2878-4 | 1 or 3 | Occipital, left nuchal plane frag. | | | | |
| 2878-8 | 3 | Mandible, left body frag. with P1 and M1 | 2878-2D | | | |
| 2878-21D | 3 | Lower left P1 | on 2878-8 | | | |
| 2878-2D | 1, 2 or 3 | Lower left P2 (isolated) | 2878-8 | X | X | |
| 2878-22D | 3 | Lower left M1 | on 2878-8 | | | |
| 2878-37 | 3 | Hand middle phalanx 2-4 | | | | |
| 2878-38 | 3 | Hand proximal phalanx 3-4, proximal extremity broken off | | | | |
| 2878-39 | 3 | Left hand proximal phalanx 5, prox. extremity broken off, distal extremity partially broken | | | | |
| C5-1 | 3 | Left parietal frag. without sutures | 2878-3 | | X | X |
| C5-2 | 3 | Lumbar vertebra 1-4, left pedicle and left superior articular process | | | | |
| C5-3 | 3 | Mandible, right ascending ramus frag. | C5-4 | | | |
| C5-4 | 3 | Mandible, right gonial angle | C5-3 | | | |
| C5-5 | 3 | Mandible, left body inferior frag. | | | | |
| C5-6 | 3 | Right temporal, squamous frag. and complete petrous | | | | |
| C5-7 | 3 | Left temporal, mastoid portion frag. | | | | |
| C5-8 | 3 | Left zygomatic, frontal process | | | | |
| Q48-1 | 2 | Left pubis superior frag. | Q376-36 | | | |
| Q53-4 | 3 | Right humerus diaphysis frag. | Humerus III | X | X | |
| Q53-5 | 2 | Ulna? diaphysis frag. | | | | |
| Q54-4 | 1 | Right tibia diaphysis frag. | Tibia III | | | |
| Q54-5 | 1 | Right tibia diaphysis frag. | Tibia IV? | | | |
| Q55-1 | 3 | Left clavicle, lateral half | | X | X | |
| Q55-3 | 3 | Right tibia diaphysis frag. | Tibia IV | | | |
| Q55-4 | 3 | Right tibia diaphysis frag. | Tibia IV | | X | X |
| Q55-5 | 3 | Left femur diaphysis frag. | Femur III | | | |
| Q55-6 | 3 | Right rib 11?, distal half | Q376-25 | | | |
| Q55-7 | 3 | Femur diaphysis frag. | | | | |
| Q56-1 | 3 | Right femur diaphysis frag. | Femur I | X | X | X |
| Q56-2 | 3 | Left tibia diaphysis frag. | Tibia I | | | |
| Q56-5 | 3 | Right tibia diaphysis frag. | Tibia III | | | |
| Q56-6 | 3 | Left femur diaphysis frag. | Femur IV | | | |
| Q56-7 | 2 | Right femur diaphysis frag. | Femur II | | | |
| Q56-8 | 3 | Right tibia diaphysis frag. | Tibia IV | | | |
| Q56-9 | 3 | Right tibia diaphysis frag. | Tibia V | | | |
| Q56-10 | 3 | Right tibia diaphysis frag. | Tibia V | | | |
| Q56-11 | 2 | Right tibia diaphysis frag. | Tibia IV | | | |
| Q56-12 | 2 or 3 | Right radius diaphysis frag. | Radius I | | | |

| | | | | | | |
|---|---|---|---|---|---|---|
| Q56-13 | 2 or 3 | Right radius diaphysis frag. | Radius I | | | |
| Q56-14 | 1 | Right humerus, diaphysis and neck frag. | Humerus III | | | |
| Q56-17 | 2 | Tibia? diaphysis frag. | | | | |
| Q57-1 | 1 | Left tibia diaphysis frag. | Tibia II | X | X | X |
| Q57-2 | 1 | Right femur diaphysis frag. | Femur II | X | X | X |
| Q57-3 | 1 | Right tibia diaphysis frag. | Tibia VI | X | X | X |
| Q98-1 | 2 | Right femur, prox. extremity frag. with lesser trochanter and diaphysis frag. | Femur I | | | |
| Q100-3 | 2 | Right rib 1, distal shaft frag. | | | | |
| Q115-1 | 3 | Left femur diaphysis frag. | Femur IV | | | |
| Q115-2 | 3 | Left femur diaphysis frag. | Femur III | | | |
| Q115-3 | 3 | Tibia or femur diaphysis frag. | | | | |
| Q116-2 | 3 | Left tibia diaphysis frag. | Tibia I | | | |
| Q116-3 | 3 | Radius, head and neck frag. | | | | |
| Q119-2 | 1 | Left rib 7? shaft frag. | Q376-7? | | X | X |
| Q305-2 | 3 | Left tibia diaphysis frag. | Tibia I | | | |
| Q305-3 | 3 | Left tibia diaphysis frag. | Tibia I | | | |
| Q305-4 | 3 | Left tibia diaphysis frag. | Tibia I | X | X | X |
| Q305-7 | 3 | Right tibia diaphysis frag. | Tibia III | | X | X |
| Q305-8 | 3 | Right tibia diaphysis frag. | Tibia VI | | | |
| Q305-11 | 3 | Tibia diaphysis frag. | | | | |
| Q305-12 | 3 | Femur diaphysis frag. | | | | |
| Q374a-1 | 3 | Right tibia diaphysis frag. | Tibia V | | X | X |
| Q375-1 | 3 | Right femur diaphysis frag. | Femur I | | | |
| Q375-2 | 3 | Left tibia diaphysis frag. | | | | |
| Q375-3 | 3 | Left tibia diaphysis frag. | Tibia I | | | |
| Q375-4 | 3 | Left tibia diaphysis frag. | Tibia I | | | |
| Q375-6 | 3 | Right tibia diaphysis frag. | Tibia IV | | | |
| Q375-7 | 3 | Right radius diaphysis frag. | Radius I | | | |
| Q375-8 | 3 | Right radius diaphysis frag. | Radius I | | | |
| Q375-9 | 3 | Femur diaphysis frag. | | | | |
| Q375-10 | 3 | Right humerus, diaphysis and neck frag. | Humerus II? | | | |
| Q376-1 | 3 | Hand proximal phalanx 2-4, both extremities broken off | | X | X | |
| Q376-2 | 3 | Right femur diaphysis frag. | Femur I | | | |
| Q376-5 | 3 | Left tibia diaphysis frag. | Tibia I | | | |
| Q376-6 | 3 | Right tibia diaphysis frag. | Tibia IV | | | |
| Q376-7 | 3 | Left rib 7? shaft frag. | Q119-2? | | | |
| Q376-8 | 3 | Left rib 1, sub-complete | | | | |
| Q376-9 | 3 | Right rib 11? shaft frag. | | | | |
| Q376-11 | 3 | Left rib 10 shaft frag. | | | | |
| Q376-12 | 3 | Left rib 4?, shaft frag. with costal angle | | | | |
| Q376-13 | 3 | Right rib 2 shaft frag. | | | | |
| Q376-14 | 3 | Left rib 4-5?, distal half/third | | | | |
| Q376-16 | 3 | Right rib 3? shaft frag. | | | | |
| Q376-17 | 3 | Left? rib 4-9? shaft frag. | | | | |
| Q376-18 | 3 | Right tibia diaphysis frag. | Tibia III? | | | |
| Q376-20 | 3 | Right humerus diaphysis frag. | Humerus II | X | X | |
| Q376-25 | 3 | Right rib 11? shaft frag. | Q55-6 | | | |
| Q376-26 | 3 | Rib 3-11 shaft frag. | | | | |
| Q376-27 | 3 | Right? rib 3-7? shaft frag. | | | | |
| Q376-28 | 3 | Left rib 5-9 shaft frag. | | | | |
| Q376-29 | 3 | Right rib 3-9 shaft frag. | | | | |
| Q376-30 | 3 | Right? rib 6-8?, sternal end | | | | |
| Q376-31 | 3 | Left? rib 11?, distal half | | | | |
| Q376-32 | 3 | Left rib 3, frag. preserving the neck, costal tubercle and angle | | | | |
| Q376-33 | 3 | Rib 8-11? shaft frag. | | | | |
| Q376-35 | 3 | Humerus head frag. | | | | |
| Q376-36 | 3 | Left pubis inferior frag. | Q48-1 | | | |

For the numbering system, see Supplementary Note S4. Element numbers (Roman numerals) were given to the most complete bones and indicate refits (column ‘Refits with / on’). º see Supplementary Note S6. Note that all of the specimens come from the RBINS collections and were excavated by E. Dupont, except for * that belongs to the RMAH material from A. de Loë’s excavations.

**Supplementary Table S2. Neandertal bones (after refitting) from the Troisième caverne of Goyet with indication of anthropogenic modifications.**

| Bone piece | Identification | Anthropogenic modifications | | | |
|---|---|---|---|---|---|
| | | Cutmarks | Percussion notches | Percussion pits | Retoucher |
| ***Craniofacial skeleton*** | | | | | |
| 2878-3 + C5-1 | Right and left parietal fragments with portion of sagittal suture | | | | |
| 2878-1 | Right parietal, postero-superior frag. | | | | |
| 2878-2 | Right parietal, anterior frag. | | | | |
| 2878-4 | Occipital, left nuchal plane frag. | | | | |
| C5-6 | Right temporal, squamous frag. and complete petrous | X? | | | |
| C5-7 | Left temporal, mastoid portion frag. | | | | |
| C5-8 | Left zygomatic, frontal process | | | | |
| 2861-1 | Right and left maxillae, alveolar and palatine processes | | | | |
| 2878-8 | Mandible, left body frag. with P1 and M1 | | | | |
| C5-3 + C5-4 | Mandible, right gonial angle & ascending ramus frag. | X | | | |
| C5-5 | Mandible, left body inferior frag. | | | | |
| ***Trunk*** | | | | | |
| C5-2 | Lumbar vertebra 1-4, left pedicle and left superior articular process | | | | |
| Q100-3 | Right rib 1, distal shaft frag. | | | | |
| Q376-8 | Left rib 1, sub-complete | X | | | |
| Q376-13 | Right rib 2 shaft frag. | | | | |
| Q376-16 | Right rib 3? shaft frag. | | | | |
| Q376-32 | Left rib 3, frag. preserving the neck, costal tubercle and angle | | | | |
| Q376-27 | Right? rib 3-7? shaft frag. | | | | |
| Q376-29 | Right rib 3-9 shaft frag. | | | | |
| Q376-30 | Right? rib 6-8?, sternal end | | | | |
| Q376-9 | Right rib 11? shaft frag. | | | | |
| Q55-6 + Q376-25 | Right rib 11? shaft | X | | | |
| Q376-26 | Rib 3-11 shaft frag. | | | | |
| Q376-12 | Left rib 4?, shaft frag. with costal angle | | | | |
| Q376-14 | Left rib 4-5?, distal half/third | | | | |
| Q376-17 | Left? rib 4-9? shaft frag. | | | | |
| Q376-28 | Left rib 5-9 shaft frag. | | | | |
| Q119-2 | Left rib 7? shaft frag. | | | | |
| Q376-7 | Left rib 7? shaft frag. | | | | |
| Q376-11 | Left rib 10 shaft frag. | X | | | |
| Q376-31 | Left? rib 11?, distal half | | | | |
| Q376-33 | Rib 8-11? shaft frag. | | | | |
| ***Upper limb*** | | | | | |
| Q55-1 | Left clavicle, lateral half | X | | | |
| Humerus II (Q376-20) | Right humerus diaphysis frag. | X | | | |
| Q375-10 (Humerus II?) | Right humerus, diaphysis and neck frag. | | | | |
| Humerus III (2 spec.) | Right humerus, diaphysis and neck frag. | | | | |
| Q376-35 | Humerus head frag. | | | | |
| Radius I (4 spec.) | Right radius diaphysis | | X | X | |
| Q116-3 | Radius, head and neck frag. | X | | | |
| Q53-5 | Ulna? diaphysis frag. | | | | |
| 2878-37 | Hand middle phalanx 2-4 | | | | |
| 2878-38 | Hand proximal phalanx 3-4, proximal extremity broken off | | | | |
| 2878-39 | Left hand proximal phalanx 5, prox. extremity broken off, distal extremity partially broken | | | | |
| Q376-1 | Hand proximal phalanx 2-4, both extremities broken off | | | | |
| ***Lower limb*** | | | | | |
| Q48-1 + Q376-36 | Left pubis | X | | | |
| Femur I (4 spec.) | Right femur diaphysis & prox. extremity frag. | X | X | X | |
| Femur II (2 spec.) | Right femur diaphysis frag. | X | X | X | |
| Femur III (3 spec.) | Left femur diaphysis frag. | X | X | X | X |
| Femur IV (2 spec.) | Left femur diaphysis frag. | X | X | X | |
| Q55-7 | Femur diaphysis frag. | | | X | |
| Q375-9 | Femur diaphysis frag. | | X | | |
| Q305-12 | Femur diaphysis frag. | | | | |

| | | | | | |
|---|---|---|---|---|---|
| Q115-3 | Tibia or femur diaphysis frag. | | | | |
| Tibia I (8 spec.) | Left tibia diaphysis | X | X | | |
| Tibia II (Q57-1) | Left tibia diaphysis frag. | X | X | | |
| Tibia III (3 spec.) | Right tibia diaphysis frag. | X | X | X | X |
| Q376-18 (Tibia III?) | Right tibia diaphysis frag. | X | | | |
| Tibia IV (6 spec.) | Right tibia diaphysis | X | X | X | X |
| Q54-5 (Tibia IV?) | Right tibia diaphysis frag. | | | | |
| Tibia V (3 spec.) | Right tibia diaphysis frag. | X | X | X | X |
| Tibia VI (2 spec.) | Right tibia diaphysis frag. | X | X | | |
| Q375-2 | Left tibia diaphysis frag. | X | | | |
| Q56-17 | Tibia? diaphysis frag. | | | | |
| Q305-11 | Tibia diaphysis frag. | | | | |

See Supplementary Fig. S8 for the placement of the anthropogenic modifications on the bones.

**Supplementary Table S3. Sample information and results of the elemental chemical analyses of the Neandertal remains from Goyet.**

| Specimen ID | $^{14}C$ dating lab # (CIO) | $\%C_{coll}$ | $\%N_{coll}$ | $C_{coll}:N_{coll}$ |
|---|---|---|---|---|
| 2878-2D | GrA-54028 | 41.4 | 14.4 | 3.4 |
| C5-1 | - | 43.0 | 14.7 | 3.4 |
| Q53-4 | GrA-54022 | 42.9 | 15.1 | 3.3 |
| Q55-1 | GrA-54257 | 36.9 | 12.9 | 3.3 |
| Q55-4 | - | 39.6 | 14.0 | 3.3 |
| Q56-1 | GrA-46170* | 45.4 | 15.5 | 3.4 |
| Q57-1 | GrA-46173* | 46.0 | 16.8 | 3.2 |
| Q57-2 | GrA-54024 | 42.7 | 15.0 | 3.3 |
| Q57-3 | GrA-60019 | 43.8 | 15.4 | 3.3 |
| Q119-2 | - | 38.9 | 13.8 | 3.3 |
| Q305-4 | GrA-46176* | 47.1 | 16.7 | 3.3 |
| Q305-7 | - | 41.9 | 14.9 | 3.3 |
| Q374a-1 | - | 43.1 | 15.2 | 3.3 |
| Q376-1 | GrA-46178* | 46.7 | 17.0 | 3.2 |
| Q376-20 | GrA-60018 | 39.8 | 14.0 | 3.3 |

* indicates collagens extracted at the CIO; all others were extracted at Tübingen University where elemental chemical analyses and stable isotope analyses (41) were also performed.

**Supplementary Table S4. Minimum number of elements (MNE) and minimum number of individuals (MNI) represented by the Goyet Neandertal bone assemblage along with the percentage representation (PR) of the elements.**

| | **MNE** | **MNI** | **PR (%)** |
|---|---|---|---|
| Cranium | 2 | 2 | 50.0 |
| Mandible | 1 | 1 | 25.0 |
| Vertebrae | 1 | 1 | 1.0 |
| Sacrum | 0 | 0 | 0.0 |
| Ribs | 11 | 2 | 11.5 |
| Sternum | 0 | 0 | 0.0 |
| Scapula | 0 | 0 | 0.0 |
| Clavicle | 1 | 1 | 12.5 |
| Humerus | 2 | 2 | 25.0 |
| Radius | 1 | 1 | 12.5 |
| Ulna | 1 | 1 | 12.5 |
| Carpus + metacarpus | 0 | 0 | 0.0 |
| Hand phalanges | 4 | 1 | 3.6 |
| Os coxae | 1 | 1 | 12.5 |
| Femur | 4 | 3 | 50.0 |
| Patella | 0 | 0 | 0.0 |
| Tibia | 6 | 4 | 75.0 |
| Fibula | 0 | 0 | 0.0 |
| Foot | 0 | 0 | 0.0 |
| *Total* | *35* | *4* | *4.9* |

The PR is calculated as MNE*100/(MNImax*NEind) with MNImax being the highest MNI for the whole sample and NEind the number of elements per individual.

**Supplementary Table S5. Faunal sample from Dupont's excavations at the Troisième caverne of Goyet identified during the present study with indication of the storage drawers and "fauna-bearing levels" (FBL).**

| | | **Q53 (FBL 2)** | **Q55 (FBL 3)** | **Q375 (FBL 3)** | **Q376 (FBL 3)** | ***Total*** |
|---|---|---|---|---|---|---|
| Perissodactyla | Horse (*Equus caballus*) | 99* | 89* | 146* | 108* | *442* |
| Artiodactyla | Reindeer (*Rangifer tarandus*) | 47* | 240* | 354 | 262 | *903* |
| | Red deer (*Cervus elaphus*) | 2 | 3 | 6 | 3 | *14* |
| | Roe deer (*Capreolus capreolus*) | | | 1 | | *1* |
| | Megaceros (*Megaloceros giganteus*) | | 2 | 3 | | *5* |
| | Bovid (*Bos primigenius* or *Bison priscus*) | 11 | 10 | 15 | 15 | *51* |
| | Ibex (*Capra ibex*) | 7 | 1 | 5 | 1 | *14* |
| | Wild boar (*Sus scrofa*) | 2 | | | 1 | *3* |
| Proboscidea | Mammoth (*Mammuthus primigenius*) | 2 | 3 | | 1 | *6* |
| Lagomorpha | Leporid (*Lepus timidus* or *Oryctolagus cuniculus*) | 1 | | 2 | 3 | *6* |
| Ungulata | Ungulate 3/4 (horse or bovid) | | 5 | | | *5* |
| | Ungulate 5 (rhinoceros or mammoth) | | | 1 | | *1* |
| Carnivora | Bear (*Ursus spelaeus* or *Ursus arctos*) | 16 | | 30 | 10 | *56* |
| | Fox (*Vulpes vulpes* or *Vulpes lagopus*) | 6 | | 5 | 1 | *12* |
| | Large canid (*Canis sp.*) | 4 | | 5 | 2 | *11* |
| | Hyaena (*Crocuta crocuta spelaea*) | 1 | 1 | 4 | 2 | *8* |
| | Badger (*Meles meles*) | 1 | | | | *1* |
| | Carnivora indet | | | 1 | | *1* |
| Mammal indet | | 2 | | | | *2* |
| Bird | | 5 | 1 | | 8 | *14* |
| *Total* | | *206* | *355* | *578* | *417* | *1556* |

* indicates the faunal specimens that were observed for the presence of anthropogenic modifications.

**Supplementary Table S6. Comparison of the horse, reindeer and Neandertal skeletal representation at Goyet.**

| | NEANDERTAL | | | | HORSE | | | | REINDEER | | | |
|---|---|---|---|---|---|---|---|---|---|---|---|---|
| | **NISP** | **NEind** | **NISP/ NEind** | **%NISP/NEind / (NISP/NEind)max** | **NISP** | **NEind** | **NISP/ NEind** | **%NISP/NEind / (NISP/NEind)max** | **NISP** | **NEind** | **NISP/ NEind** | **%NISP/NEind / (NISP/NEind)max** |
| Cranium | 9 | 1 | 9 | 64.29 | 24 | 2 | 12 | 21.43 | 12 | 2 | 6 | 4.67 |
| Mandible | 4 | 1 | 4 | 28.57 | | | | | | | | |
| Cervical vertebrae | 0 | 7 | 0 | 0 | 4 | 86 | 0.05 | 0.08 | 0 | 64 | 0 | 0 |
| Thoracic vertebrae | 0 | 12 | 0 | 0 | | | | | | | | |
| Lumbar vertebrae | 1 | 5 | 0.2 | 1.43 | | | | | | | | |
| Sacrum | 0 | 1 | 0 | 0 | | | | | | | | |
| Ribs | 21 | 24 | 0.88 | 6.25 | | | | | | | | |
| Scapula | 0 | 2 | 0 | 0 | 14 | 2 | 7 | 12.5 | 2 | 2 | 1 | 0.78 |
| Clavicle | 1 | 2 | 0.5 | 3.57 | N/A | | | | N/A | | | |
| Humerus | 5 | 2 | 2.5 | 17.86 | 39 | 2 | 19.5 | 34.82 | 101 | 2 | 50.5 | 39.30 |
| Radius | 5 | 2 | 2.5 | 17.86 | 74 | 2 | 37 | 66.07 | 116 | 2 | 58 | 45.14 |
| Ulna | 1 | 2 | 0.5 | 3.57 | | | | | | | | |
| Coxal | 2 | 2 | 1 | 7.14 | 5 | 2 | 2.5 | 4.46 | 4 | 2 | 2 | 1.56 |
| Femur | 14 | 2 | 7 | 50 | 96 | 2 | 48 | 85.71 | 137 | 2 | 68.5 | 53.31 |
| Tibia | 28 | 2 | 14 | 100 | 112 | 2 | 56 | 100 | 257 | 2 | 128.5 | 100 |
| Fibula | 0 | 2 | 0 | 0 | 0 | 2 | 0 | 0 | 0 | 2 | 0 | 0 |
| Carpus+tarsus+patella | 0 | 32 | 0 | 0 | 3 | 28 | 0.11 | 0.19 | 4 | 24 | 0.17 | 0.13 |
| Metacarpus+metatarsus | 0 | 20 | 0 | 0 | 64 | 12 | 5.33 | 9.52 | 259 | 12 | 21.58 | 16.80 |
| Phalanges | 4 | 56 | 0.07 | 0.51 | 5 | 12 | 0.42 | 0.74 | 6 | 48 | 0.13 | 0.10 |
| Indet | 1 | - | - | - | 2 | - | - | - | 5 | - | - | - |
| *Total* | *96* | *177* | *0.54* | *3.87* | *442* | *132* | *3.35* | *5.98* | *903* | *151* | *5.98* | *4.65* |

NISP: Number of identified specimens, NEind: Number of elements per individual, (NISP/NEind)max: maximal value of NISP/NEind. Variable definitions are after ref. 42. The horse and reindeer specimens were identified among a sample of Dupont's collection from FBL 2 and 3 (Supplementary Table S5).

**Supplementary Table S7. Palaeogenetic results of the mtDNA reads for the 10 Goyet Neandertal specimens analysed before and after postmortem damage (PMD) filtering.**

**Before filtering (NoPMD)**

| Sample ID | Library | Unique mapping reads | Average coverage of mtDNA (fold) | Nucleotides covered at 5-fold coverage (% of mtDNA) | Average read length (base pairs) | Damage at 5' end (%) | Polluting fragments | Clean fragments | Contaminaton estimate (95 % confidence interval) |
|---|---|---|---|---|---|---|---|---|---|
| C5-1 | Sample_MA130m | 957 | 5.56 | 9445 (57.00) | 96.21 | 7 | 137 | 86 | 61.4 (54.9–67.6) % |
| Q55-4 | Sample_MA124m | 1964 | 9.60 | 14838 (89.57) | 81.01 | 20 | 85 | 351 | 19.5 (16.0–23.5) % |
| Q56-1 | Sample_GA71 | 11682 | 44.93 | 16555 (99.94) | 63.70 | 32 | 58 | 1715 | 3.3 (2.5–4.2) % |
| Q57-1 | Sample_GA72 | 6096 | 24.19 | 16257 (98.14) | 65.73 | 35 | 137 | 819 | 14.3 (12.3–16.7) % |
| Q57-2 | Sample_GA73 | 12905 | 49.91 | 16564 (99.99) | 64.06 | 39 | 166 | 1886 | 8.1 (7.0–9.3) % |
| Q57-3 | Sample_GA66 | 29758 | 94.92 | 16538 (99.84) | 52.83 | 39 | 167 | 3452 | 4.6 (4.0–5.3) % |
| Q119-2 | Sample_MA131m | 1025 | 4.94 | 8465 (51.11) | 79.79 | 14 | 75 | 138 | 35.2 (29.1–41.8) % |
| Q305-4 | Sample_GA74 | 36399 | 146.25 | 16565 (100.00) | 66.55 | 39 | 136 | 5739 | 2.3 (2.0–2.7) % |
| Q305-7 | Sample_MA127m | 10690 | 46.68 | 16561 (99.98) | 72.33 | 26 | 45 | 2147 | 2.1 (1.5–2.7) % |
| Q374a-1 | Sample_MA125m | 15796 | 69.85 | 16562 (99.99) | 73.24 | 34 | 12 | 3124 | 0.4 (0.2–0.7) % |

**After filtering (PMD)**

| Sample ID | Library | Unique mapping reads | Average coverage of mtDNA (fold) | Nucleotides covered at 5-fold coverage (% of mtDNA) | Average read length (base pairs) | Damage at 5' end (%) | Polluting fragments | Clean fragments | Contaminaton estimate (95 % confidence interval) |
|---|---|---|---|---|---|---|---|---|---|
| C5-1 | Sample_MA130m | 187 | 0.96 | 282 (1.71) | 91.72 | 20 | 8 | 24 | 25.0 (13.3–42.1) % |
| Q55-4 | Sample_MA124m | 613 | 2.69 | 2740 (16.55) | 75.07 | 46 | 5 | 90 | 5.3 (2.3–11.7) % |
| Q56-1 | Sample_GA71 | 4096 | 14.53 | 14925 (90.11) | 58.77 | 67 | 5 | 506 | 1.0 (0.4–2.3) % |
| Q57-1 | Sample_GA72 | 2142 | 7.77 | 11119 (67.12) | 60.09 | 64 | 9 | 264 | 3.3 (1.7–6.1) % |
| Q57-2 | Sample_GA73 | 4741 | 16.77 | 16109 (97.25) | 58.60 | 71 | 4 | 601 | 0.7 (0.3–1.7) % |
| Q57-3 | Sample_GA66 | 12387 | 38.51 | 16011 (96.66) | 51.50 | 71 | 16 | 1312 | 1.2 (0.7–1.9) % |
| Q119-2 | Sample_MA131m | 223 | 0.93 | 126 (0.77) | 76.00 | 28 | 9 | 43 | 17.3 (9.4–29.7) % |
| Q305-4 | Sample_GA74 | 13980 | 52.37 | 16520 (99.73) | 62.04 | 70 | 9 | 1986 | 0.5 (0.2–0.9) % |
| Q305-7 | Sample_MA127m | 3784 | 15.60 | 16333 (98.60) | 71.04 | 60 | 5 | 690 | 0.7 (0.3–1.7) % |
| Q374a-1 | Sample_MA125m | 6922 | 28.68 | 16522 (99.75) | 71.17 | 65 | 1 | 1228 | 0.1 (0.0–0.5) % |

**Supplementary Table S8. Palaeogenetic results of the mtDNA reads for the three low coverage specimens mapped against modern human and Neandertal mitochondrial reference sequences before postmortem damage filtering.**

| Sample ID | Library | Unique mapping reads | | Average coverage of mtDNA (fold) | | Nucleotides covered at 5-fold coverage (% of mtDNA) | | Average read length (base pairs) | |
|---|---|---|---|---|---|---|---|---|---|
| | | *Modern human* | *Neandertal* | *Modern human* | *Neandertal* | *Modern human* | *Neandertal* | *Modern human* | *Neandertal* |
| C5-1 | Sample_MA130m | 996 | 957 | 5.87 | 5.56 | 9950 (60.05) | 9445 (57.00) | 97.69 | 96.21 |
| Q55-4 | Sample_MA124m | 1899 | 1964 | 9.17 | 9.60 | 14550 (87.85) | 14838 (89.57) | 80.01 | 81.01 |
| Q119-2 | Sample_MA131m | 1037 | 1025 | 4.97 | 4.94 | 8504 (51.34) | 8465 (51.11) | 79.45 | 79.79 |

## Supplementary Notes

## Supplementary Note S1. The Troisième caverne of Goyet and its regional context

The Goyet caves are located in Mozet, Belgium, some 20 km from the well-known site of Spy (Supplementary Fig. S1). The Troisième caverne of Goyet (50°26’44”N, 5°00’48”E) is part of a large karstic system developed in a Carboniferous limestone cliff of the Ardenne Massif some 130 m above sea level on the right bank of the Samson Valley, a tributary of the Meuse. The main Goyet caves open onto a large terrace about 15 m above the river. The Troisième caverne, the archaeologically richest of the Goyet cave system, is about 120 meters deep and consists of three chambers (1, 2). Chamber A lies at the entrance of the cave and is connected to Chamber B by a small gallery, with Chamber C situated at the back of the cave. Edouard Dupont, the main excavator of the site, distinguished four “fauna-bearing levels” or FBL in Chamber A (2). He recovered a significant quantity of Middle and Upper Palaeolithic artefacts, numerous Pleistocene mammal bones, especially herbivores, and human remains from the uppermost three FBL (ref. 3; see also Supplementary Note S3). Many of these bones were marrow cracked, have cutmarks, or bear traces of ochre (4, 5). At the rear of Chamber A and in Chamber B, Dupont (2) distinguished a fourth and fifth FBL containing mainly cave bear, cave lion, and cave hyaena remains. The faunal material from these levels appears unrelated to the anthropogenic assemblages from the front of Chamber A given the presence of numerous carnivore traces and comparatively less human-modified material (3, 4). The mammal assemblage from Chamber C contains remains of, amongst others, cave bear, cave hyaena, horse, reindeer, as well as human skeletal material (3, 6) that might not be of Palaeolithic age (see “Stratigraphic provenience” in Supplementary Note S4).

Interestingly, E. Dupont considered the possibility of cannibalism at Goyet in his unpublished handwritten notes from 1906 recently discovered by one of us (M.G). Concerning the human remains from Chamber A, Dupont wrote:

> […] all of the Caves and their archaeological levels contained some human remains mixed with those of consumed animals. The consistency of the evidence leads us to conclude cannibalism. And, indeed, in the 3rd level, adult and adolescent: cutmarks on an ulna; 2nd level, two adults and adolescent: cutmarks on a clavicle; 1st level, adult and adolescent: cutmarks on a skull fragment (our translation).

However, the two human remains we were able to match with Dupont’s description (fragments of a clavicle and parietal) are not Neandertal but rather modern human remains. Nearly all of the Goyet Neandertal material was identified during our work (Supplementary Notes S3 and S4).

Very few Northern European sites north of 50° N have yielded MIS 3 Neandertal remains (Supplementary Fig. S1). In Germany, the remains of at least three individuals identified at the Neandertal type-site have been attributed to this group along with a partial parietal bone from Warendorf (7). While several other German sites have yielded Middle Palaeolithic human remains, their age is still too uncertain to be considered here (7). This is also the case with an isolated frontal bone fragment recovered from the North

Sea (8). Further east, a small number of isolated teeth have been recently identified at Stajnia Cave (Poland; 9). In Belgium, the Trou de l'Abîme at Couvin and Walou Cave at Trooz have each yielded an isolated tooth, whereas the remains of two adults and one juvenile were discovered at Spy (10).

## Supplementary Note S2. Assessment of the Goyet Middle Palaeolithic material and overview of the Late Mousterian from the Mosan Basin

The lack of field data from Dupont's excavations at the Troisième caverne makes it impossible to determine whether the typo-technologically Middle Palaeolithic material corresponds to one or several occupations. The latter possibility was suggested in the only, albeit incomplete study of this material by Ulrix-Closset (11), basing her conclusion on the diversity of surface alterations and several typological arguments. Ulrix-Closset noted that Levallois technology is poorly represented, with *débitage* involving primarily "spherical cores." Various scrapers forms are the most abundant retouched tool type, followed by 45 Mousterian points and the occasional limace. She also stressed the presence of bifacial scrapers alongside 28, often 'atypical' bifacial tools, three of which resemble foliate pieces (11). The numerous denticulates and raclettes present in the assemblage are more appropriately interpreted as edge-damaged artefacts, highlighting the significant post-depositional reworking of the deposits. Ulrix-Closset (11) concluded that the majority of the assemblage could be assigned to the Quina Mousterian ("Charentian") mixed with a smaller bifacial component representing either the Mousterian of Acheulean Tradition or an "evolved Mousterian." The latter was tentatively identified as the final phase of the Mosan Mousterian marked by foliate bifacial pieces (but see ref. 12 for a critical review of the "evolved Mousterian").

Several similarly dated late Middle Palaeolithic sites in the Mosan Basin provide insights concerning the Goyet lithic assemblage. At Scladina, the material from layer 1A contains evidence for different flake production systems similar to the Levallois, Quina and discoid methods that are adapted to the local raw materials (13). The assemblage from layer CI-8 of Walou Cave comprises primarily unifacial and Levallois *débitage* accompanied by a scraper-rich retouched tool component, including one Mousterian point (14). The material from Trou de l'Abîme at Couvin is made on a fine-grained, non-local flint that was heavily reduced. Scrapers are the most well-represented tool type and occur alongside small bifacial pieces (15).

## Supplementary Note S3. Reassessment of the Goyet collections

In 2008, we began the revision of the Goyet human collections and systematic sorting of the faunal material from the Troisième caverne in order to identify any overlooked human remains. Focusing on the Dupont collections, and more specifically, a series of 21 drawers each measuring approximately 75 cm x 54 cm and containing "indeterminate" fauna (Supplementary Fig. S2), all material possibly associated with the Troisième caverne for which we had access was reassessed by two biological anthropologists (H.R and I.C; see Methods). Given the fragmentary nature of the human remains identified in these drawers, as well as clear anthropogenic marks indicating non-taphonomic (i.e. intentional), post-mortem fragmentation, the faunal collections were entirely resorted in order to isolate any additional skeletal fragments with similar

taphonomic features and morphometric characteristics (e.g. cortical thickness, medullar morphology). The refitting of specimens securely identified as human during the first sorting with non-diagnostic fragments isolated during this second phase confirmed the identification of the latter as human. Several of these newly identified human specimens were then selected for direct radiocarbon ($^{14}C$) dating. A palaeontological and taphonomic study of a sample of fragmentary, "indeterminate" fauna from the Dupont collection was also carried out. Additionally, human remains were sampled for stable isotope and genetic analysis, and additional samples were selected for dating based on the results of the previous analyses. The "indeterminate" fauna from Dupont’s excavations was sorted a third time by another biological anthropologist (A.G.-O) who identified several additional human remains. Refits were attempted again to maximize the number of Neandertal remains.

At the start of the project, the collections held by the Anthropology and Prehistory Section of the Royal Belgian Institute of Natural Sciences (RBINS) included 70 bone specimens and 33 isolated teeth from Dupont’s excavations at the Troisième caverne identified as human. In addition to identifying numerous new human remains among the faunal collections, our analysis excluded six bone fragments and a single tooth erroneously identified as human. The human remains from the Troisième caverne of Goyet now comprise 244 bone specimens and 39 isolated teeth. The remains of at least 16 individuals (nine adults/adolescents and seven juveniles) can be associated with levels 1 through 4 and represent a mix of materials from different periods. A fragment of human tibia from level 3 was previously dated to 1,985 +/- 70 years BP (OxA-5678) (16) and we identified human remains from three different periods of the Upper Palaeolithic amongst the material from levels 1 to 3 (17). Moreover, we identified 96 bone specimens and three isolated teeth that we attribute to Neandertals (Supplementary Table S1 and Supplementary Notes S4 and S5) and which are the focus of the present contribution.

## Supplementary Note S4. Labelling and provenience of the Goyet Neandertal remains

**Labelling system.** The Neandertal specimens were labelled using several codes that reflect their recent research history. Some of the Neandertal remains (nine bone specimens and two isolated teeth) were already identified as human by E. Dupont and labelled 2878 or 2861 in his catalogue. These labels were subsequently amended with an additional number to individualize each specimen, probably when Fr. Twiesselmann was head of the Anthropology and Prehistory Section of the RBINS (Jadin, pers. com.). The remains numbered 2878 were labelled in red ink, with the additional number later added in black ink. The fact that the human remains numbered 2861 bear both numbers in black ink probably reflects their not having been originally labelled at the time of Dupont. We know that the numbers from Dupont’s catalogue were lost before Twiesselmann arrived at the RBINS and that he asked one of his technicians to try to find the correspondence between Dupont’s numbering system and the materials housed at the RBINS (Jadin, pers. com.). The numbers in black probably date from this time.

The remains labelled Qxxx-xx and Cx-x are specimens that we identified among the fauna recovered by E. Dupont from the Troisième caverne. Qxxx and Cx indicate the fauna drawers in which the human remains were found, and each of the specimens was individualized by adding a dash and a number after the

drawer number. These drawers are currently housed in two different reserves of the Palaeontology Section of the RBINS. Finally, one human specimen (1189-1) was identified in a drawer (no. 1189) of the archaeology collection held by the Anthropology and Prehistory Section of the RBINS.

A single tooth (1424-3D) was found amongst the material held by the Royal Museums of Art and History (RMAH, Brussels) from A. de Loë's early 20th century excavations at Goyet. Its identification number (1424) follows the RMAH inventory system for the portion of the Goyet collection it comes from. This was the third human tooth (3D) we isolated from this collection. Note that the ID of each human tooth from both the RBINS and RMAH includes a "D" (*dent* being French for tooth) to clearly differentiate them from the human bones.

**Stratigraphic provenience.** E. Dupont stated having discovered human remains in the upper four FBL of the Troisième caverne (2). The provenience of most of the Neandertal remains was indicated on small yellow labels glued to the bone fragments. While the remains numbered 2878 lack such labels, unpublished notes from the end of the 19th century kept in the RBINS archives contain a list of the remains labelled 2878 as well as the FBL from which they were recovered (i.e. FBL 1, 2 or 3). Comparison of the brief description of the remains in these notes with those labelled 2878 allowed us to correlate most of the remains with their FBL. Only a single Neandertal bone labelled 2861 (maxilla 2861-1) proved problematic. Dupont indicated in his unpublished handwritten notes that the remains numbered 2861 come from FBL 4 in Chamber C of the Troisième caverne and were found together with the remains of cave hyaena. Chamber C lies more than 100 m from Chamber A, where all the other Neandertal remains are supposed to originate, including the upper lateral incisor 2878-1D that refits with 2861-1 (Supplementary Table S1). Moreover, the latter shows a very different aspect from the other human remains labelled 2861, which are fresh and partially eroded, pointing to a different taphonomic history. We believe that maxilla 2861-1 may have been misplaced with the 2861 material after the excavations but before the remains numbered 2878 were inventoried and labelled at the end of the 19th century.

## Supplementary Note S5. The Neandertal assemblage from the Troisième caverne

**Identification**. The Neandertal remains from the Troisième caverne were isolated from the rest of the human sample on the basis of their morphometric characteristics combined with their taphonomic aspect, isotopic ratios, radiocarbon dating and genetic analysis (see Table 1 and Supplementary Tables S1 and S3). Distinguishing morphometric traits include, the mastoid development on temporal C5-6; evidence for several missing wormian bones on the parietal fragment 2878-1; posterior position of the mental foramen on mandible 2878-8 and presence of the "horizontal-oval" type mandibular foramen on C5-3; crown and root morphology of the teeth; development of the manual phalanx extremity relative to the diaphysis; curvature of the diaphysis of radius I and constant height of the articular edge of the head of radius fragment Q116-3 along its circumference; length of the superior ramus of pubis Q48-1; presence of a developed gluteal buttress and a well-developed lesser trochanter on femur I; short length of the diaphysis of tibia I relative to its overall dimensions.

**Age-at-death.** The Neandertal sample is relatively homogeneous both in terms of size and robustness. Most of the Neandertal bone pieces are too poorly preserved to securely determine whether they are fully mature. However, they are all of adult size and compatible with an age-at-death during adolescence or adulthood. No modifications associated with senescence are visible. The stage of development and attrition of the dental material associated with maxilla 2861-1 (including the isolated upper left lateral incisor 2878-1D) and mandible 2878-8 (including the isolated lower left second premolar 2878-2D) is also compatible with an age-at-death during adolescence or young adulthood. The third isolated tooth (lower left lateral incisor 1424-3D) has an open root apex (stage A½ after ref. 18) pointing to an age-at-death between ca. 6.5 and 12.5 years according to modern standards (18, 19), making it the youngest Neandertal individual of the sample.

**Minimum Number of Individuals (MNI).** The Neandertal sample includes a child (see above) as well as several adolescent or adult individuals based on the representation and morphometrics of the tibia combined with the mtDNA analysis. Right tibias are represented by six pieces and left tibias by three (Supplementary Fig. S3).

- The three most complete right tibias (tibias III, IV and V) each represent a different individual as they all overlap in the area of the soleal line.
- Right tibia VI, a distal portion of the anterior diaphysis, not only has a different surficial aspect compared to the other right tibias but it also produced a different mtDNA sequence to those obtained from tibias III, IV and V. It thus represents a fourth individual. Left tibia II, a proximal portion of the anterior diaphysis, shares the same surficial aspect as right tibia VI as well as an identical mtDNA sequence for all covered positions (ca. 98 % of the mtDNA). Additionally, the morphometric characteristics of these two tibias are compatible with them belonging to the same individual. With the data at hand, tibia II thus does not represent an additional individual.
- The most complete tibia of the Neandertal sample, left tibia I, cannot be the antimere of tibias III, V or VI as its mtDNA sequence differs from those obtained for these bones nor can it be associated with left tibia II as they both preserve the area of the tibial tuberosity in addition to carrying different mtDNA sequences. Unfortunately, the mtDNA of tibia IV is not sufficiently preserved to determine whether it carried the same mtDNA sequence as that of tibia I. The morphometric characteristics and taphonomic aspect of the two bones are, however, compatible with their being corresponding antimeres, and we cannot exclude that they belong to the same individual.
- The remaining tibia specimens (Q54-5 –a proximal portion of right anterior diaphysis, Q375-2 –a proximal portion of left posterior diaphysis, and Q376-18 –a long portion of right anterior crest) are all compatible with belonging to at least one of the tibial elements identified above or their antimere, and thus cannot presently be attributed to additional individuals.

The Goyet Neandertal collection represents a minimum of five individuals: at least four different adolescent or adult individuals alongside the child represented by a single tooth (the lower left lateral incisor 1424-3D).

**Individual associations.** The number of elements from the Neandertal collection for which individual associations can be securely proposed is limited given the fragmentary nature of the collection. Left and right

tibias II and VI may belong to the same individual based on morphometric, taphonomic and genetic similarities (see above). They also share identical mtDNAs for all covered positions (ca. 98 % of the mtDNA) with femur II whose morphometric and taphonomic characteristics are compatible with it belonging to the same individual, hence we tentatively associated them. Femur I shares an identical mtDNA sequence with right tibias III and V, and its morphometric and taphonomic characteristics are compatible with it belonging to the same individual as one of these tibias. Finally, we tentatively associated left and right tibias I and IV based on morphometric and taphonomic similarities (see above).

## Supplementary Note S6. Radiocarbon dating of the Goyet Neandertals

Radiocarbon dates are reported in years before present (BP) following the convention proposed by Mook and van der Plicht (20). These dates require calibration to obtain calendar ages. The presently recommended calibration curve is IntCal13 (21), and calibration was done using the OxCal software (version 4.2; ref. 22). Calibrated ages are reported in calBP, defined as calendar age relative to 1950 AD.

The first attempt at dating the Goyet Neandertal material concerned the roots of two teeth (2861-26D, the upper right P1 of maxilla 2861-1, and 2878-2D, the lower left P2 of mandible 2878-8). However, collagen was not extracted and the dates (GrA-46009: 27,070 +160, -150 BP and GrA-46010: 18,090 +80, -70 BP) obtained from dentine powder were too young given that the morphometric characteristics of the teeth securely identify them as Neandertal. Material being still available for 2878-2D, collagen extracted from this tooth was re-dated. Unfortunately, the second date (GrA-54028) also came back too young (Table 1), possibly due to undetected contamination (Supplementary Table S3). Tooth 2878-2D was part of the sample identified as human by E. Dupont and, although unnoticed during sampling, it may have been varnished like the rest of the human bones in Dupont’s collection.

The dates obtained on the Goyet Neandertal bones range from 36,590 +300, -270 to 41,200 +500, -410 BP, or from 40.6 to 45.6 ky calBP at 2 sigmas. Although the $^{14}C$ dates of specimens Q57-1, Q57-2 and Q57-3 span the whole range of dates obtained for the Neandertal sample (Table 1), their morphology, taphonomy and mtDNA do not preclude their belonging to the same individual. Moreover, anthropogenic modifications on the Neandertal remains reflect similar behaviours as well as being located in the same position across the assemblage (see “Taphonomic analysis of the Goyet Neandertal material and anthropogenic modifications” section). These different lines of evidence suggest undetected contamination as the most likely explanation for the youngest $^{14}C$ ages, in which case the Goyet Neandertal sample would represent a single chronological group dated to ca. 44–45.5 ky calBP. In the absence of definitive evidence, we propose a conservative range of ca. 40.5–45.5 ky calBP for the Goyet Neandertals.

## Supplementary Note S7. Palaeogenetic analyses of the Goyet Neandertals

Each bone fragment selected for palaeogenetic analysis was first irradiated with UV light in order to reduce surface DNA contamination. A dental drill was used to remove a thin layer of bone surface and to

sample inside the bone. An aliquot of between 30 mg and 120 mg of bone powder was utilized in the DNA extraction following an optimized protocol to retrieve typical short ancient DNA (aDNA) fragments (23). 10–20 µl out of 100 µl of extract were transformed into a sequencing library using a double stranded library preparation protocol (24) and indexed with an individual double index combination (25). Different amplification cycles were used for each indexed sequencing library in order to avoid heteroduplex products formation. Mitochondrial DNA (mtDNA) was subsequently enriched through a bead-capture protocol that uses modern human mtDNA fragments as baits (26). The enriched libraries were re-amplified, quantified on a DNA 1000 chip (Agilent), pooled in equal concentration with other samples, and sequenced on an Illumina HiSeq 2500 rapid run (2x101+8+8 cycles).

The sequenced reads were quality filtered and merged using established protocols (27). Only merged reads with a length above 30 base pairs (bp) were mapped against a Neandertal mitochondrial reference sequence using BWA (28) in combination with an in-house developed circular mapping tool (29). After removal of identical reads appearing more than once, sequences with a mapping quality score lower than 30 were excluded using the SAMtools software package (30). Only unique sequences securely placed within the mtDNA (Supplementary Table S7) were used to reconstruct the mitochondrial consensus sequence of each sample for positions with at least 5-fold coverage using the custom iterative assembler MIA (31).

In order to authenticate taxonomic assignment, reads of the three low coverage samples (C5-1, Q55-4 and Q119-2) were mapped against the modern human mitochondrial reference sequence (rCRS). The mapping results are in the same range as the values obtained using the Neandertal reference (Supplementary Table S8) therefore excluding a reference bias in the taxonomic assignment. Moreover, potential contamination with modern human DNA was assessed with a contamination estimation software that considers positions where the Neandertal mtDNA reference sequence differs from at least 99 % of 311 worldwide modern human mtDNAs (31). For each of these diagnostic positions, we compared the number of sequences matching the Neandertal reference better (clean fragments) than modern human mtDNAs (polluting fragments) to calculate contamination (Supplementary Table S7). Moreover, the characteristic damage pattern of aDNA was calculated as the percentage of reads showing C to T or G to A misincorporations respectively at the 5' and 3' ends of DNA fragments (Supplementary Table S7 and Supplementary Fig. S17) using a program first used in Briggs *et al.* (32).

The phylogenetic placement of the seven newly generated complete or almost complete mitochondrial sequences (i.e. at least 98 % complete) was assessed by comparing them to modern human, Neandertal and Denisovan mitochondrial genomes. The MUSCLE software (33) was used to align mtDNA consensus sequences of the Goyet specimens, 54 modern humans belonging to different worldwide language groups (34), eight Neandertals (31, 35–37), and one Denisovan individual (38). A maximum parsimony tree and a maximum likelihood tree with complete deletions (16,110 positions considered) and 1,000 iterations as bootstrap support were built using MEGA 5.2 (39) and refined with FigTree (ref. 40; Fig. 2 and Supplementary Fig. S4).

Subsequently, reads with nucleotide misincorporations (postmortem damage or PMD score ≥ 3)

indicating authentic ancient origin (Supplementary Table S7) were selected with PMD tools (37). The percentage of filtered fragments with damaged termini increased up to 71 % whereas contamination decreased for all samples (Supplementary Table S7). Only the filtered reads of the Goyet samples represented in Fig. 2 were used to generate new mitochondrial consensus sequences (for positions covered at least 5 times) that were aligned to the same Neandertal mtDNA reference and assembled in additional maximum parsimony and maximum likelihood trees (Supplementary Figs. S18 and S19) using the same parameters mentioned above (10,234 positions considered). This confirmed the phylogenetic placement of the original reconstructed mitochondrial consensus sequences within the Neandertal mtDNA diversity and validated the intragroup matrilineal relationships.

## Supplementary Information References


1. van den Broeck, E., Martel, E. A. & Rahir, E. *Les Cavernes et les Rivières Souterraines de la Belgique Etudiées Spécialement dans leurs Rapports avec l'Hydrologie des Calcaires et avec la Question des Eaux Potables. Tome 2: Les Calcaires Carbonifériens du Bassin de Dinant et Coup d'Oeil sur le Bassin de Namur* (H. Lamertin, 1910).
2. Dupont, E. *L'Homme Pendant les Ages de la Pierre dans les Environs de Dinant-sur-Meuse, 2ème Edition* (C. Muquardt Ed., 1872).
3. Germonpré, M. A reconstruction of the spatial distribution of the faunal remains from Goyet, Belgium. *Notae Praehistoricae* **21,** 57–65 (2001).
4. Germonpré, M. Preliminary results on the mammals of the Magdalenian upper horizon of Goyet (Belgium). *Notae Praehistoricae* **16,** 75–85 (1996).
5. Germonpré, M. & Hämäläinen, R. Fossil bear bones in the Belgian Upper Paleolithic: The possibility of a proto-bear-ceremonialism. *Arctic Anthropol.* **44,** 1–30 (2007).
6. Germonpré, M. & Sablin, M. V. The cave bear (*Ursus spelaeus*) from Goyet, Belgium. The bear den in Chamber B (bone horizon 4). *Bull. Inst. R. Sc. N. B.* **71,** 209–233 (2001).
7. Street, M., Terberger, T. & Orschiedt, J. A critical review of the German Paleolithic hominin record. *J. Hum. Evol.* **51,** 551–579 (2006).
8. Hublin, J.-J. *et al.* Out of the North Sea: the Zeeland Ridges Neandertal. *J. Hum. Evol.* **57,** 777–785 (2009).
9. Nowaczewska, W. *et al.* The tooth of a Neanderthal child from Stajnia Cave, Poland. *J. Hum. Evol.* **64,** 225–231 (2013).
10. Pirson, S. *et al.* Chronostratigraphic context of the Middle to Upper Palaeolithic transition: Recent data from Belgium. *Quat. Int.* **259,** 78–94 (2012).
11. Ulrix-Closset, M. *Le Paléolithique Moyen dans le Bassin Mosan en Belgique* (Universa, 1975).
12. Flas, D. *La Transition du Paléolithique Moyen au Supérieur dans la Plaine Septentrionale de l'Europe* (Anthropologica et Praehistorica 119, 2008).
13. Di Modica, K. *Les Productions Lithiques du Paléolithique Moyen en Belgique: Variabilité des Systèmes d'Acquisition et des Technologies en Réponse à une Mosaïque d'Environnements Contrastés*. Thèse de doctorat (MNHN, Université de Liège, 2010).
14. Draily, C. *La Grotte Walou à Trooz (Belgique). Fouilles de 1996 à 2004. Volume 3: L'Archéologie* (Etudes et Documents – Archéologie 22, Institut du Patrimoine Wallon, 2011).
15. Toussaint, M. *et al.* The Neandertal lower right deciduous second molar from Trou de l'Abîme at Couvin, Belgium. *J. Hum. Evol.* **58,** 56–67 (2010).
16. Bronk Ramsey, C., Higham, T. F. G., Owen, D. C., Pike, A. W. G. & Hedges, R. E. M. Radiocarbon dates from the Oxford AMS System: Datelist 31. *Archaeometry* **44(3), Suppl 1,** 1–149 (2002).
17. Rougier, H. *et al.* The first Upper Paleolithic human remains from Belgium: Aurignacian, Gravettian and Magdalenian fossils at the "Troisième caverne" of Goyet. *PaleoAnthropology* **2013,** A 33 (2013).
18. Moorrees, C. F. A., Fanning, E. A. & Hunt, E. E. Age variation of formation stages for ten permanent teeth. *J. Dent. Research* **42,** 1490–1502 (1963).
19. AlQahtani, S. J., Hector, M. P. & Liversidge, H. M. Brief communication: The London atlas of human tooth development and eruption. *Am. J. Phys. Anthrop.* **142,** 481–490 (2010).
20. Mook, W. G. & van der Plicht, J. Reporting $^{14}$C activities and concentrations. *Radiocarbon* **41,** 227–239 (1999).
21. Reimer, P. J. *et al.* IntCal13 and Marine13 radiocarbon age calibration curves 0-50,000 years cal BP. *Radiocarbon* **55,** 1869–1887 (2013).
22. Bronk Ramsey, C. Bayesian analysis of radiocarbon dates. *Radiocarbon* **51,** 337–360 (2009).
23. Dabney, J. *et al.* Complete mitochondrial genome sequence of a Middle Pleistocene cave bear reconstructed from ultrashort DNA fragments. *Proc. Natl. Acad. Sci. USA* **110,** 15758–15763 (2013).
24. Meyer, M. & Kircher, M. Illumina sequencing library preparation for highly multiplexed target capture and sequencing. *Cold Spring Harb. Protoc.* **6,** pdb.prot5448 (2010).
25. Kircher, M., Sawyer, S. & Meyer, M. Double indexing overcomes inaccuracies in multiplex sequencing on the Illumina platform. *Nucleic Acids Res.* **40,** e3 (2012).
26. Maricic, T., Whitten, M. & Pääbo, S. Multiplexed DNA sequence capture of mitochondrial genomes using PCR products. *PLoS ONE* **5,** e14004 (2010).
27. Kircher, M. in *Ancient DNA. Methods and Protocols*. (eds Shapiro, B. & Hofreiter, M.) 197–228 (Humana Press, 2012).
28. Li, H. & Durbin, R. Fast and accurate short read alignment with Burrows-Wheeler transform. *Bioinformatics* **25,** 1754–1760 (2009).
29. Peltzer, A. *et al.* EAGER: efficient ancient genome reconstruction. *Genome Biol.* **17,** 60 (2016).
30. Li, H. *et al.* The sequence alignment/map format and SAMtools. *Bioinformatics* **25,** 2078–2079 (2009).
31. Green, R. E. *et al.* A complete Neandertal mitochondrial genome sequence determined by high-

throughput sequencing. *Cell* **134,** 416–426 (2008).
32. Briggs, A. W. *et al.* Patterns of damage in genomic DNA sequences from a Neandertal. *Proc. Natl. Acad. Sci. USA* **104,** 14616–14621 (2007).
33. Edgar, R. C. MUSCLE: multiple sequence alignment with high accuracy and high throughput. *Nucleic Acids Res.* **32,** 1792–1797 (2004).
34. Ingman, M., Kaessmann, H., Pääbo, S. & Gyllensten, U. Mitochondrial genome variation and the origin of modern humans. *Nature* **408,** 708–713 (2000).
35. Briggs, A. W. *et al.* Targeted retrieval and analysis of five Neandertal mtDNA genomes. *Science* **325,** 318–321 (2009).
36. Prüfer, K. *et al.* The complete genome sequence of a Neanderthal from the Altai Mountains. *Nature* **505,** 43–49 (2014).
37. Skoglund, P. *et al.* Separating endogenous ancient DNA from modern day contamination in a Siberian Neandertal. *Proc. Natl. Acad. Sci. USA* **111,** 2229–2234 (2014).
38. Krause, J. *et al.* The complete mitochondrial DNA genome of an unknown hominin from southern Siberia. *Nature* **464,** 894–897 (2010).
39. Tamura, K. *et al.* MEGA5: molecular evolutionary genetics analysis using maximum likelihood, evolutionary distance, and maximum parsimony methods. *Mol. Biol. Evol.* **28,** 2731–2739 (2011).
40. Rambaut, A. *FigTree v1. 3.1: Tree figure drawing tool*. (2009) Available at: http://tree.bio.ed.ac.uk/software/figtree/.
41. Wißing, C. *et al.* Isotopic evidence for dietary ecology of Late Neandertals in North-Western Europe. *Quat. Int.* doi: 10.1016/j.quaint.2015.09.091 (2015).
42. Lyman, R. L. Quantitative units and terminology in Zooarcheology. *Am. Antiq.* **59,** 36–71 (1994).
43. Sawyer, G. J. & Maley, B. Neanderthal reconstructed. *Anat. Rec. B New Anat.* **283B,** 23–31 (2005).
44. Barone, R. *Anatomie Comparée des Mammifères Domestiques, Tome I Ostéologie – Atlas* (Vigot, 1976).
45. Fontana, L. Mobilité et subsistance au Magdalénien dans le Bassin de l'Aude. *Bull. Soc. Préhist. Fr.* **96,** 175–190 (1999).
